# The Historical Biogeography of Divergence in the Relict Cypress *Chamaecyparis obtusa*, and the Implications for Conservation and Management in East Asia

**DOI:** 10.1002/ece3.72240

**Published:** 2025-09-30

**Authors:** Takaki Aihara, Chih‐Hsin Cheng, Chiou‐Pin Chen, Chieh‐Ting Wang, Kentaro Uchiyama, Daiki Takahashi, Yoshihisa Suyama, Yoshihiko Tsumura

**Affiliations:** ^1^ Institute of Life and Environmental Sciences University of Tsukuba Tsukuba Ibaraki Japan; ^2^ School of Forestry and Resource Conservation National Taiwan University Taipei Taiwan; ^3^ The Experimental Forest, College of Bio‐Resources and Agriculture National Taiwan University Taipei Taiwan; ^4^ Department of Forest Molecular Genetics and Biotechnology Forestry and Forest Products Research Institute Tsukuba Ibaraki Japan; ^5^ Faculty of Agriculture Kyushu University Fukuoka Japan; ^6^ Kawatabi Field Science Center, Graduate School of Agricultural Science Tohoku University Sendai Miyagi Japan

**Keywords:** *Chamaecyparis*, conifer, disjunct distribution, genetic diversity, population genetics, relict plant species

## Abstract

East Asia provides long‐term stable refugia for relict plant species and supports high species richness. *Chamaecyparis obtusa* is a typical relict species that is now restricted to particularly warm, humid areas in East Asia, mainland Japan, and Taiwan. It is widely used for timber, and understanding its genetic characteristics within its natural range is important for appropriate management and conservation. This study used genome‐wide single nucleotide polymorphisms (SNPs) to examine the historical biogeography as well as genetic characteristics of *C. obtusa* populations across its distribution range. High levels of genetic divergence were found between mainland Japan and Taiwan (0.673–0.717 *F*_ST_). The initial divergence occurred around 1 million years ago (Ma) based on a neighbor‐joining tree and 1.32 Ma (with a 95% confidence interval of 0.20–2.54 Ma) based on a DIYABC analysis, during the early Pleistocene when the land bridge connecting mainland Japan and Taiwan collapsed. Populations in mainland Japan exhibited higher genetic diversity, suggesting frequent gene flow and past population expansions. Within mainland Japan, both northern and southern marginal populations exhibited high levels of genetic distinctness and are considered to represent past refugia from the last glacial period. The populations in Taiwan exhibited lower genetic differentiation, even though infrequent gene flow was seen between them. All the *C. obtusa* populations studied exhibited random mating based on *F*_IS_ values, and continuous conservation of restricted areas is indicated. The highly divergent populations emphasize the need for conservation, and seedling transfers between the different genetic clusters identified are not recommended.

## Graphical abstract

High levels of genetic divergence exist between *C. obtusa* in Japan and Taiwan. Divergence is estimated to have occurred around 1 Ma, during the early Pleistocene. Populations in Japan show higher genetic diversity than those in Taiwan. Populations in Japan suggest frequent gene flow and past population expansions. Marginal populations in Japan display high levels of genetic distinctness.

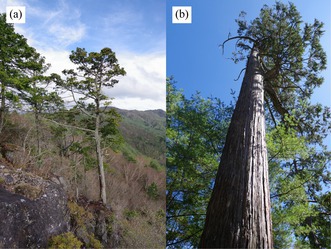

## 1 Introduction

East Asia (EA) was generally not covered by ice sheets during the glacial periods of the Quaternary (2.6 million years ago (Ma)–present), allowing a greater persistence and higher species richness of vascular plants compared with other areas at the same latitude, such as North America (NA) and Europe (Kryshtofovich 1929; Latham and Ricklefs 1993; Qian and Ricklefs 2000). Accordingly, EA has provided long‐term stable refugia for relict plant species (Qian and Ricklefs 2000; Manchester et al. 2009; Tang et al. 2018). Paleogene–Neogene relict species (referred to as Tertiary relict species) were distributed across a large part of the Northern Hemisphere during the Paleogene and Neogene, but today are restricted to limited areas. These species often show disjunct distribution patterns in the warm and wet climatic conditions that prevail over NA, southwest Eurasia, and EA (Milne and Abbott 2002). In EA, Paleogene–Neogene relict species can be divided into two distinct groups, ‘Pacific track’ pattern and ‘Atlantic track’ pattern, which reflect differences in divergence time and migration routes (Milne and Abbott 2002). Within the ‘Pacific track’ pattern, plant taxa that are distributed disjunctively in Japan and NA are thought to have migrated via the Bering land bridge (BLB) before it was broken (4.8–5.5 Ma; Marincovitch Jr. and Gladenov 1999).

*Chamaecyparis* (Cupressaceae) is a relict genus; fossils have been found across vast areas of the Northern Hemisphere, from the Paleogene and Neogene (Liu et al. 2009), and exhibit a typical EA–NA disjunct distribution (Farjon 2005). Within this genus, *C. obtusa* was thought to be a relict species in EA that had migrated via the BLB because a sister species exists in NA, *C. lawsonia* (Wang et al. 2022). *Chamaecyparis obtusa* is now restricted to particularly warm humid areas in EA, mainland Japan ( *C. obtusa* s.s.; in this study, referred to as *C. obtusa* for convenience) and Taiwan ( *C. obtusa* var. *formosana*). *Chamaecyparis obtusa* var. *formosana* in Taiwan is distinguished from *C. obtusa* in mainland Japan by its smaller and finer foliage and much smaller cones (Bailety 1914). It is known that more than 100 plant species are distributed across both mainland Japan and Taiwan (Hsieh 2003), even though these land masses have not been connected directly via the Ryukyu islands since the early Pleistocene (Ota 1998). The process of divergence in plant taxa between mainland Japan and Taiwan could be key to understanding the plant richness and refugia of relict species in EA.

Previous studies have suggested that *C. obtusa* in Japan and *C. obtusa* var. *formosana* in Taiwan diverged 1.3 Ma (Wang et al. 2003), and *C. obtusa* var. *formosana* is nested in the clade of *C. obtusa* (Liao et al. 2010). It therefore appears that the species migrated from mainland Japan to Taiwan (Huang 2014), although only limited sample numbers in Japan have been studied. However, *Quercus glauca* and *Trochodendron aralioides*, which also distribute disjunctively in mainland Japan, the Ryukyu islands, and Taiwan, exhibit higher genetic diversity in Taiwan than in Japan (Huang et al. 2002, 2004), suggesting that the Taiwanese populations are ancestral. Natural *C. obtusa* populations do not exist today in the Ryukyu islands, but the phylogeographic history of *C. obtusa* can be re‐visited using modern molecular methods with samples from across the entire species range. Differences in the results of genetic diversity and structure of *C. obtusa* populations within Japan have been observed between molecular markers (Uchida et al. 1997; Tsumura et al. 2007; Matsumoto et al. 2010). Therefore, analyses of the genetic characteristics of *C. obtusa* using genome‐wide molecular methods and abundant samples would be informative. Next‐generation sequencing (NGS) technology (Metzker 2010), which has been developed over the last decade, offers an effective genotyping system for population biology, and deciphering regional population demographies and range shifts using NGS could provide a deeper insight into the biogeographical history of target species.

**FIGURE 1:**
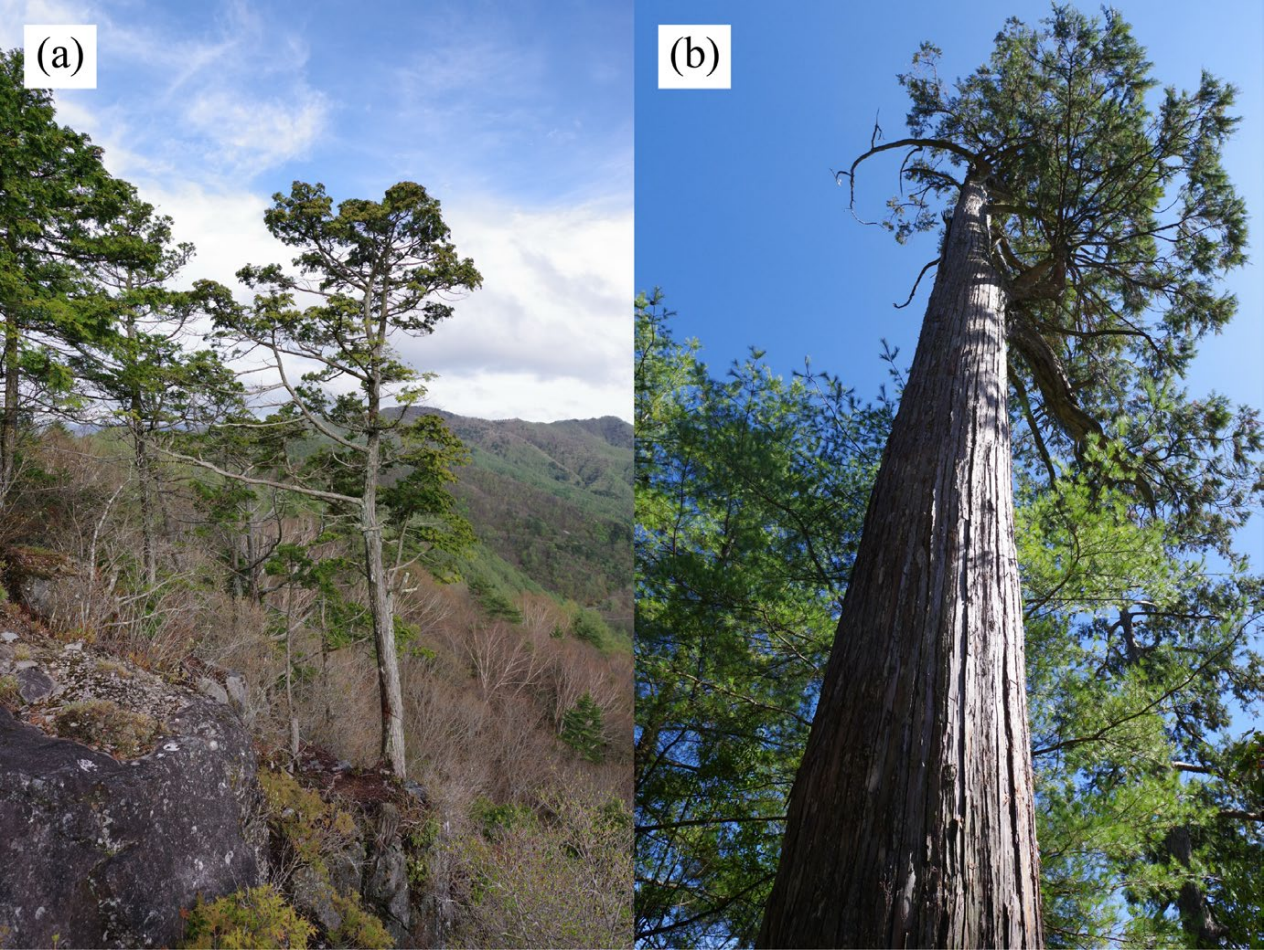
*Chamaecyparis obtusa* in Japan (a) (May 2025 in Chichibu) and *C. obtusa* var. *formosana* in Taiwan (b) (November 2023 in Heping District).

*Chamaecyparis obtusa* is important for its horticultural value; it is characterized by a pleasant scent, a very light, almost white color, strength, and resistance to humidity and decay. In Japan, the timber of this species has been used for house construction as well as temple and shrine construction since at least the Nara era (AD 710; Suzuki 2002). Both the amount of timber products and plantation area of *C. obtusa* in Japan are the second largest, after *Cryptomeria japonica* , representing about 3176 thousand m^3^ (about 6.5% of the net timber production) and 2568 thousand ha (about 6.8% of the national land area) in 2024 (Japan Forest Agency 2024). In Japan today, natural forests of this species are scattered in isolated locations (Figure 1a) ranging from the Fukushima Prefecture (37°10′ N) in northern Japan to Yakushima Island (30°15′ N) in southern Japan. In Taiwan, *C. obtusa* var. *formosana* grows in the northern and central cloud forests at an altitude between 1500 and 2500 m a.s.l. (Forestry and Nature Conservation Agency 2023), and includes huge trees that are more than 1000 years old (Figure 1b). Japan used to import large high‐quality timber from natural forests in Taiwan for its own temple and shrine construction, before the Taiwanese government prohibited the logging of natural forests in 1990 (Editorial Committee of History of Precious Woods 1986; Mineo and Matsushita 2021). Elucidating the genetic properties of such natural forest populations is crucially important for the conservation and appropriate management of *C. obtusa* .

In this study, we aimed to examine the genetic characteristics of different populations across the whole species range of *C. obtusa* and *C. obtusa* var. *formosana*, and infer the populations' demographic history and any potential change in distribution since the last glacial maximum (LGM) by using molecular methods to analyze genome‐wide regions. Finally, by looking at the divergence of the populations of *C. obtusa* and *C. obtusa* var. *formosana*, we discussed how they are restricted to those regions and the conservation and management of this species.

## 2 Materials and Methods

### 2.1 Sample Collection and DNA Extraction

**FIGURE 2:**
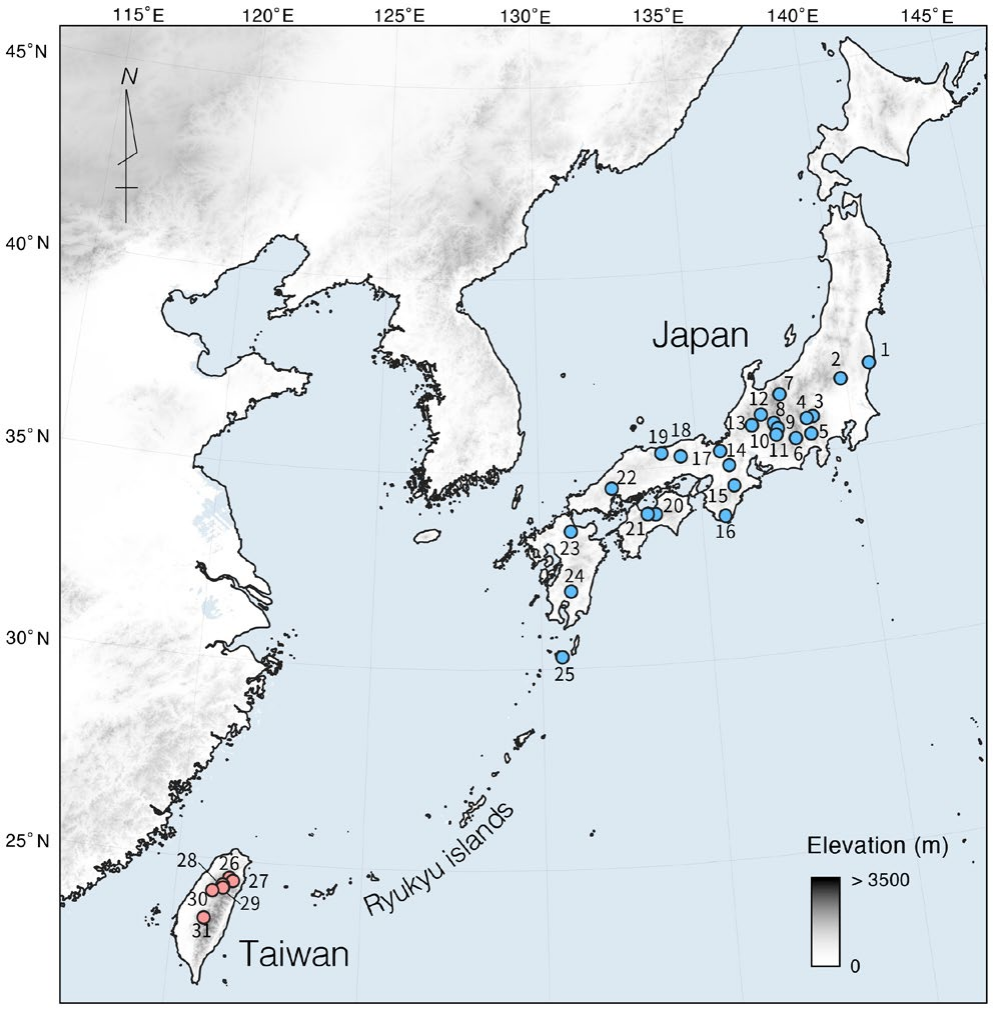
The distribution of the study populations of *Chamaecyparis obtusa* . The blue circles indicate the locations of sampling sites in Japan ( *C. obtusa* ). The red points indicate the locations of sampling sites in Taiwan ( *C. obtusa* var. *formosana*).

Samples of *C. obtusa* were collected from across its range in Japan and Taiwan. From Japan, DNA samples of 289 individuals had previously been collected from 25 natural populations (Tsumura et al. 2007). From Taiwan, in 2023, fresh leaves of *C. obtusa* var. *formosana* were collected from 100 individuals representing six natural populations (Figure 2; Table 1). Most of the sampled individuals in Taiwan represented old and giant trees (such as Cilan) or remnant forest (such as Taipinshan) and were thought to provide a reliable representation of the natural population. However, 12 individuals from the Alishan population were excluded from subsequent analyses because they appeared to have originated from a *C. obtusa* plantation in Japan, based on pre‐analysis of single nucleotide polymorphisms (SNPs). Total genomic DNA was extracted from the leaves following a modified 2× cetyltrimethylammonium bromide (CTAB) protocol (Murray and Thompson 1980).

**TABLE 1:** The study populations of *Chamaecyparis obtusa* and their genetic characteristics.

No.	Population	Abbreviation	Region	N	Latitude	Longitude	Elevation	MAT	AP	*P*	*H*_O_	*H*_E_	*F*_IS_	π	Tajima's *D*
1	Iwaki	IWK	Japan	10	37.08	140.82	106	13	1388	17	0.074	0.076	0.017	0.0026	0.100
2	Yaita	YIT	Japan	12	36.78	139.83	367	12	1560	20	0.079	0.084	0.023	0.0028	−0.201
3	Chichibu	CCB	Japan	12	35.92	138.78	1371	6	1798	0	0.083	0.087	0.026	0.0029	−0.481
4	Mizugaki	MIZ	Japan	12	35.88	138.58	1555	6	1761	0	0.079	0.081	0.018	0.0028	−0.473
5	Aokigahara	AOK	Japan	12	35.48	138.67	1010	10	1743	5	0.076	0.080	0.022	0.0029	−0.398
6	Hijiri	HIJ	Japan	12	35.42	138.17	1923	5	2221	3	0.081	0.084	0.021	0.0028	−0.434
7	Ohmachi	OMT	Japan	10	36.58	137.82	1226	9	1447	0	0.076	0.080	0.025	0.0027	−0.448
8	Ohtaki	OHT	Japan	12	35.77	137.58	1242	7	1915	3	0.076	0.080	0.025	0.0027	−0.455
9	Ontake	ONT	Japan	11	35.87	137.53	1675	6	2020	3	0.079	0.081	0.016	0.0027	−0.443
10	Akazawa	AKS	Japan	12	35.72	137.63	1202	8	1857	0	0.079	0.081	0.017	0.0027	−0.427
11	Shizumo	SZM	Japan	10	35.57	137.57	752	12	1892	0	0.077	0.079	0.019	0.0027	−0.412
12	Miyamura	MIY	Japan	11	36.12	137.15	938	10	1945	2	0.080	0.087	0.033	0.0026	−0.422
13	Aburazaka	ABZ	Japan	12	35.87	136.83	710	9	2201	6	0.084	0.085	0.017	0.0027	−0.332
14	Tanakamiyama	TNK	Japan	10	34.92	135.98	479	13	1703	0	0.077	0.080	0.024	0.0028	−0.439
15	Iitaka	ITK	Japan	12	34.38	136.08	1103	11	2419	5	0.077	0.079	0.018	0.0028	−0.429
16	Kozakawa	KOZ	Japan	11	33.63	135.72	196	15	2729	1	0.078	0.079	0.017	0.0026	−0.371
17	Ashiu	ASH	Japan	13	35.30	135.73	602	12	1955	0	0.082	0.086	0.026	0.0026	−0.491
18	Yamazaki	YMZ	Japan	11	35.25	134.48	981	9	1957	3	0.077	0.079	0.020	0.0027	−0.329
19	Misasa	MIS	Japan	10	35.37	133.88	198	12	1812	0	0.078	0.083	0.027	0.0027	−0.428
20	Shiragayama	SRG	Japan	9	33.82	133.58	1134	12	2095	2	0.074	0.077	0.021	0.0027	−0.323
21	Besshiyama	BES	Japan	12	33.83	133.33	1087	9	2380	1	0.074	0.080	0.030	0.0026	−0.416
22	Tsutsuga	TTG	Japan	10	34.53	132.27	775	11	1952	0	0.074	0.078	0.024	0.0027	−0.365
23	Hikosan	HIK	Japan	12	33.48	130.95	724	11	2469	0	0.073	0.078	0.027	0.0026	−0.489
24	Kobayashi	KBY	Japan	12	31.95	130.90	1124	11	3356	13	0.070	0.075	0.024	0.0025	−0.287
25	Yakushima	YKS	Japan	11	30.30	130.58	1084	13	3468	54	0.072	0.079	0.031	0.0027	0.098
26	Lalashan	LLS	Taiwan	2	24.73	121.43	1383	8	3295	0	0.067	0.048	0.004	0.0022	0.320
27	Taipinshan	TPS	Taiwan	11	24.50	121.53	1594	13	2758	14	0.063	0.071	0.035	0.0024	0.067
28	Cilan	CLN	Taiwan	18	24.39	121.26	2005	17	2659	22	0.060	0.071	0.039	0.0025	0.057
29	Heping District	HPD	Taiwan	19	24.23	121.00	2242	14	2744	67	0.055	0.072	0.059	0.0024	−0.024
30	Yuzhuowan	YZW	Taiwan	2	23.90	121.12	2500	13	2628	1	0.061	0.046	0.011	0.0022	0.494
31	Alishan	ALS	Taiwan	6	23.54	120.84	2403	16	2534	14	0.056	0.057	0.021	0.0021	0.272
	Japan			281				10.02	2081.72	5.52	0.077	0.081	0.023	0.0027	−0.364
	Taiwan			58				13.45	2769.67	19.67	0.060	0.061	0.028	0.0023	0.198
	All			339				11.73	2425.69	8.26	0.074	0.077	0.024	0.0026	−0.255

*Note:* The values of MAT and AP were extracted from the WorldClim 1.4 dataset (www.worldclim.org; Hijmans et al. 2005).

Abbreviations: π, nucleotide diversity; AP, annual precipitation (mm); *F*_IS_, fixation index; *H*_E_, expected heterozygosity; *H*_O_, observed heterozygosity; MAT, mean annual temperature (°C); *N*, number of examined samples after short nucleotide polymorphism (SNP) filtering steps; *P*, number of private SNPs.

### 2.2 Genome‐Wide SNP Analysis and Population Genetic Analyses

Multiplexed inter‐sample sequence repeat genotyping by sequencing (MIG‐seq; Suyama and Matsuki 2015; Suyama et al. 2022) was used to obtain genome‐wide SNPs. Library preparation for the MIG‐seq analysis was performed as described by Suyama et al. (2022), and the sequencing was conducted on an Illumina MiSeq platform (Illumina, San Diego, CA, USA) using the MiSeq Reagent Kit v3 (150‐cycle) (Illumina).

The SNPs used for genetic analyses were prepared following the protocol of Suyama et al. (2022). Sequence primers for MIG‐seq were trimmed using Trimmomatic v.0.33 (Bolger et al. 2014). A dDocent pipeline (Puritz et al. 2014) was used for quality trimming (Trimmomatic v.0.33) and read mapping (BWA mem v.0.7.12) (Li and Durbin 2009) for the whole genome sequence of *C. obtusa* (Shirasawa et al. 2024). SNPs were extracted using the populations program in Stacks v2.66 (Rochette et al. 2019), and the minimum proportion of individuals across the populations required to process a locus was set to 80% for the dataset. To remove paralogous loci and possible polymerase chain reaction (PCR) errors, we excluded loci showing an observed heterozygosity > 0.6 and eliminated SNPs with a minor allele count < 3 for all datasets. In addition, we excluded nine samples with a missing rate > 0.5. Overall, we obtained 9420 SNPs from 339 individuals.

Indices of genetic diversity, such as the number of private SNPs (*P*), observed heterozygosity (*H*_O_), expected heterozygosity (*H*_E_), and the fixation index (*F*_IS_) of each population, were calculated for variant sites using the populations program in Stacks. Nucleotide diversity (π) was calculated for all sites using the populations program. Pairwise genetic differentiation (*F*_ST_) between populations was calculated using Nei (1987) in the Hierfstat package v.0.5.11 (Goudet 2005) in R version 4.3.1 (R Core Team 2023); this does not take intra‐populational variation into account because some populations only had two samples. Based on the *F*_ST_ values for the populations, we performed a principal coordinate analysis (PCoA) using the cmdscale() function in R.

Spatial genetic structure was assessed by testing the significance of isolation by distance (IBD) using a Mantel test, with 1000 random permutations of the relationship between the matrix of pairwise *F*_ST_/(1 − *F*_ST_) and that of the geographical distance between populations. The geographical distance between populations was calculated using the function distGeo in the geosphere package v.1.5.20 (Hijmans et al. 2024). The Mantel test was carried out using the program GenoDive v.3.0 (Meirmans 2020).

To confirm inter‐ and intraspecific divergence, phylogenetic relationships between populations were inferred using a neighbor‐joining (NJ) method based on *F*_ST_. *Chamaecyparis formosensis*, sampled in Heping district, Taiwan, was selected as the outgroup. We conducted SNP calling, including one *C. formosensis* sample, with the filtering steps as mentioned above, and obtained 9667 SNPs from 340 individuals for the phylogenetic analysis. An NJ tree was inferred from BIONJ (Gascuel 1997), using algorithms to deal with incomplete distance matrices (Criscuolo and Gascuel 2008), implemented in the ape package v.5.8 (Paradis and Schliep 2019) in R. The NJ tree was visualized using the ape package and SplitsTree software version 3 (Huson and Bryant 2006) to display incomplete lineage sorting between populations. Divergence times among each node were estimated by penalized and maximum likelihoods, which assumed auto‐correlated substitution rates (Paradis 2013), using the function chronos (Sanderson 2002; Kim and Sanderson 2008) and node dating (Jones and Poon 2016) in the ape package in R.

The phylogenetic relationships between individuals were inferred from a maximum likelihood search under the GTR + ASC + G4 model using IQ‐TREE v.1.6.12 (Nguyen et al. 2015); based on the SNPs shared by 90% of individuals, 4943 SNPs were converted by vcf2phylip (Ortiz 2019). The phylogenetic tree was visualized using FigTree v.1.4.4.

Clustering analysis was conducted using the software ADMIXTURE (Alexander et al. 2009) by assuming between one and 12 ancestral populations (*K*), terminating the process when the log‐likelihood change between iterations fell below 0.0001. Cross‐validation error estimation was used to assess the most suitable value of *K*. As the clustering analysis model assumed linkage equilibrium among markers (Alexander et al. 2009), we excluded 3937 SNPs with high values of linkage disequilibrium (*R* ^2^ > 0.4) using Plink v.1.9 (Chang et al. 2015) for the ADMIXTURE analysis. Replicate runs were aligned and visualized with the pophelper package (Francis 2017) in R.

### 2.3 Demographic Inferences

To infer the past demographic history of *C. obtusa* , we analyzed a variety of parameters and conducted simulations. First, to estimate the past population demography, we calculated Tajima's *D* statistic (Tajima 1989) for each population using VCFtools v.0.1.16 (Danecek et al. 2011). A negative Tajima's *D* value is indicative of a recent selective sweep or population expansion, while a positive value suggests an excess of common variation in a region, which can be consistent with balancing selection or population contraction.

Second, to explore the divergence time and past effective population size changes of *C. obtusa* , we utilized approximate Bayesian computation algorithms (Beaumont et al. 2002). These analyses were carried out using DIYABC random forest software version 1.0 (Collin et al. 2021), renowned for its user‐friendly interface and its ability to compare the likelihood of multiple complex historical models. DIYABC facilitates the estimation of various demographic parameters and the identification of significant demographic events (Cabrera and Palsbøll 2017). We focused on the divergence between *C. obtusa* in mainland Japan and *C. obtusa* var. *formosana* in Taiwan, and identifying which one was the ancestor. The Yakushima population was set as an independent cluster to exclude high divergence rates among the Japanese clusters. We simulated six scenarios for three populations, as shown in Figure S1, with 4081 SNPs, excluding the missing loci for all Taiwanese samples and minor allele frequencies < 0.05. The best scenario was determined by the maximum value of voting (Collin et al. 2021). The vote was conducted for the most plausible scenario model for each 10,000 random forest simulations. Posterior probabilities of simulations were only shown for the best scenario in the DIYABC random forest. To convert the time scale from generations to years, we assumed 50 years per generation, as in a demographic inference of Cupressaceae, where the estimated bottleneck period coincided with the glaciation (Ma et al. 2019).

### 2.4 Species Distribution Modeling

To estimate the climatic niches of *C. obtusa* and hindcast their potential distributions during the LGM (about 22,000 years ago) and the Mid‐Holocene (MH; about 6000 years ago), we used species distribution modeling. The general modeling approach was to calibrate the distribution models using the data for current species localities and climatic variables, evaluate their predictive ability in terms of the modern distribution, and then project the models onto LGM and MH climatic data. We used maximum entropy principle algorithms (MaxEnt) (Phillips et al. 2006) with the dismo package in R for the modeling.

**FIGURE 3:**
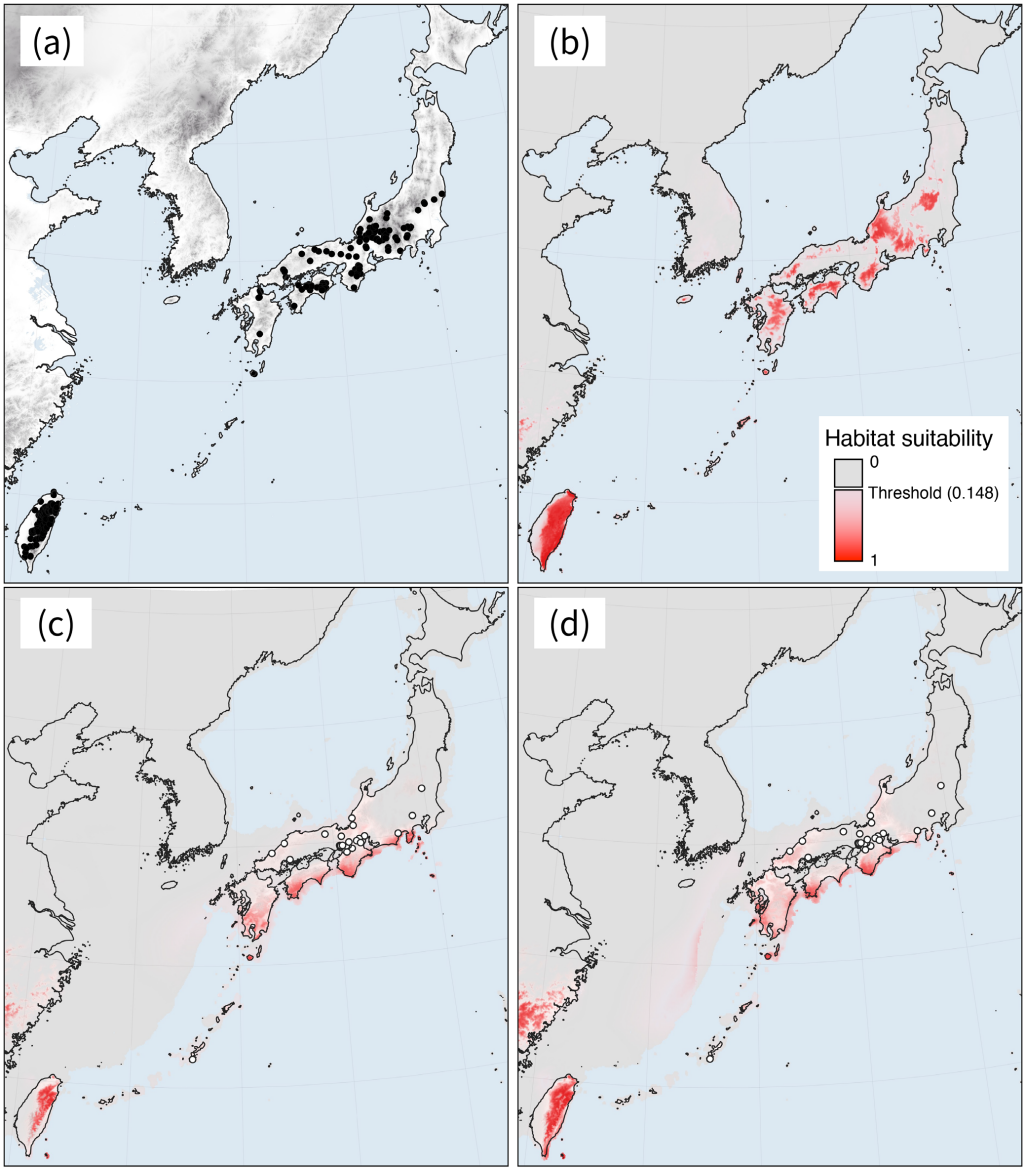
The potential distributions of *Chamaecyparis obtusa* . (a) The localities of *C. obtusa* used for species distribution modeling are indicated by black dots. The habitat suitability of *C. obtusa* under (b) current conditions and during the last glacial maximum (LGM) under the (c) MIROC‐ESM (Watanabe et al. 2011) and (d) CCSM4 (Gent et al. 2011) scenarios is shown by red shading. The white dots in (c) and (d) indicate the location of macro‐fossil records dating from around the LGM (Table S2).

The occurrence localities of *C. obtusa* in Japan were extracted from the national vegetation survey database (Ministry of the Environment 2021), in addition to the data from the 25 sampled locations. The national vegetation survey database records the main species for every height layer at 66,840 locations across Japan from the year 2000 (based on when we downloaded the data). From this database, we extracted the localities of *C. obtusa* that were thought to represent natural stands, for use as occurrence localities for *C. obtusa* in Japan. This resulted in 114 localities with natural stands. For occurrence localities of *C. obtusa* var. *formosana* in Taiwan, we used the 262 data points from the Taiwan Biodiversity Network (2024), which contains the occurrence localities of every species in each 10 km × 10 km grid (Level 2 of the TWGrid‐WGS84 Taiwan Longitude and Latitude Grid System), recorded by specimen, national inventory survey, or otherwise. To reduce spatial autocorrelation of whole occurrence data, duplicate records of occurrence localities were removed, and the data thinned at a 2.5 arcminute (ca. 5 km^2^ grid) resolution using Q GIS 3.28 (QGIS.org 2021), resulting in presence data for 248 locations (Figure 3a; Table S1). Absence data randomly created 10,000 locations in areas 5 km away from the presence data.

We adopted an approach by Austin (2007) and selected explanatory variables whose ecological processes are understood in multiple cool‐temperate tree species, including conifer species in Japan such as *Abies* species (Tanaka et al. 2012), *Tsuga* species (Tsuyama et al. 2014, 2015), *Cryptomeria japonica* (Tsumura et al. 2020), and *Thuja standishii* (Worth et al. 2021). We used four bioclimatic variables at a 2.5 arcminute spatial resolution downloaded from the WorldClim 1.4 dataset (www.worldclim.org; Hijmans et al. 2005): Bio 6, the mean daily minimum temperature of the coldest month; Bio 10, the mean temperature of the warmest quarter; Bio 18, the precipitation during the warmest quarter; and Bio 19, the precipitation during the coldest quarter. We tested the correlations among these variables (Figure S2) and confirmed that there is no strong collinearity (Menard 2002: Pearson's correlation coefficient *r* < |0.80|).

To evaluate the model's predictive ability, we employed k‐fold cross‐validation with tenfold partitioning. The presence data partitioned the testing (20%) and training (80%) data, and five MaxEnt predictions were undertaken. The MaxEnt prediction mean area under the curve (AUC) for receiver operating characteristic (ROC) analysis was used as an index of the model's predictive ability. The threshold of potential distributions of *C. obtusa* was applied to the maximum true positive rate plus the true negative rate. Potential distributions of *C. obtusa* in the LGM and MH were projected using two global climate models (GCMs) because of uncertainty in the LGM climate data, namely the Model for Interdisciplinary Research on the Climate Earth System Model (MIROC‐ESM; Watanabe et al. 2011) and the Community Climate System Model Version 4 (CCSM4; Gent et al. 2011). In addition to the potential distributions, we collected macro‐fossil records from the LGM (Table S2) to verify the modeling. The potential distributions and macro‐fossil records of *C. obtusa* were mapped using Q GIS 3.28.

In addition to estimating the whole species' potential distributions, we modeled *C. obtusa* in Japan and *C. obtusa* var. *formosana* in Taiwan separately to understand differences in climatic niches between them. After predicting the potential distribution of each using the methods described above, we compared the climatic conditions above the thresholds for each other.

## 3 Results

### 3.1 Genetic Diversity

**FIGURE 4:**
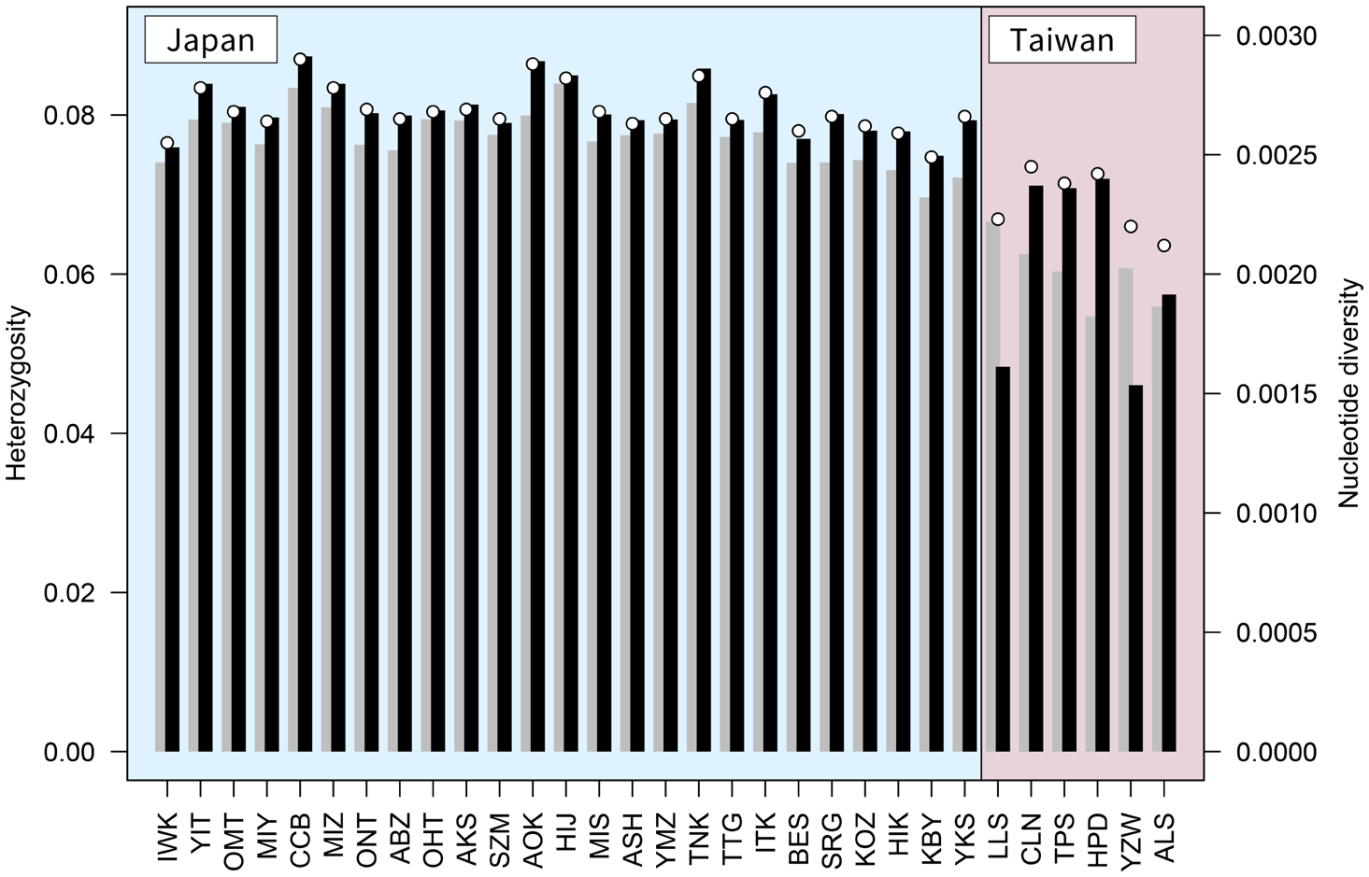
Genetic diversity of *Chamaecyparis obtusa* populations. The gray bars indicate the observed heterozygosity (*H*_O_), the black bars the expected heterozygosity (*H*_E_), and the white circles the nucleotide diversity (π), of each *C. obtusa* population. Abbreviations for the populations (Table 1) are indicated at the bottom of each panel, with higher latitudinal populations on the left side and lower latitudinal populations on the right side.

Observed heterozygosity (*H*_O_), expected heterozygosity (*H*_E_), and nucleotide diversity (π) were higher in the populations in Japan (mean *H*_O_ 0.077, *H*_E_ 0.081, π 0.0027) than those in Taiwan (mean *H*_O_ 0.060, *H*_E_ 0.061, π 0.0023) (Figure 4; Table 1) (*H*_O_: *t* = 8.93, *p* < 0.001, *H*_E_: *t* = 4.02, *p* = 0.009, π: *t* = 6.65, *p* < 0.001). The northern‐most and southern‐most populations within Japan, apart from Yakushima, displayed relatively lower expected heterozygosity and nucleotide diversity (Iwaki: *H*_E_ 0.076, π 0.0026; Kobayashi: *H*_E_ 0.075, π 0.0025) than central populations in Japan (mean *H*_E_ 0.081, π 0.0027) (Figure 4) (*H*_E_: *t* = 7.17, *p* < 0.01, π: *t* = 5.20, *p* = 0.036). Similarly, in Taiwan, the northern‐most and southern‐most populations displayed relatively lower nucleotide diversity (Lalashan: π 0.0022, Alishan: π 0.0021) than other populations in Taiwan (mean π 0.0024) (Figure 4) (*t* = 5.20, *p* = 0.095). All populations except those with low sample numbers (Lalashan and Yuzhuowan) had values *H*_O_ <*H*_E_. The mean number of private SNPs (*P*) was larger in Taiwan (19.67) than Japan (5.52) (Table 1). In Japan, the northern‐most (Iwaki and Yaita) and southern‐most (Kobayashi and Yakushima) populations had more private SNPs than other populations. All populations had fixation index (*F*_IS_) values slightly larger than 0 (Table 1).

### 3.2 Genetic Differentiation

**FIGURE 5:**
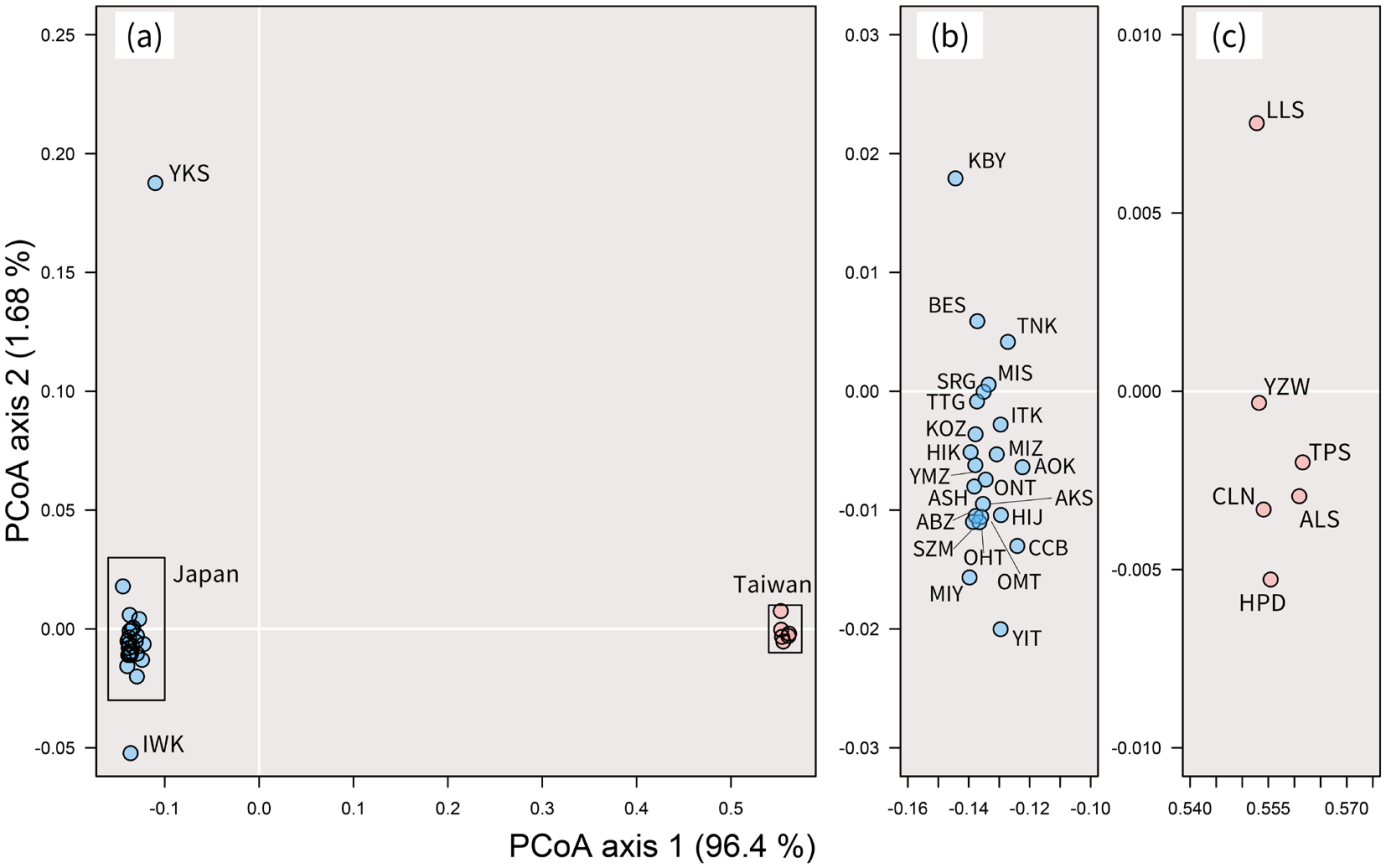
A principal coordinate analysis (PCoA) plot based on pairwise genetic differentiation (*F*_ST_). Blue circles indicate the position of populations in Japan, while red circles indicate the position of populations in Taiwan. Abbreviations for the populations (Table 1) are indicated at each panel. Plots in Japan excluding Yakushima and Iwaki are shown in (b) and plots in Taiwan are shown in (c).

The mean genetic differentiation (*F*_ST_) of populations between Japan and Taiwan was 0.694 (Table 2). The mean *F*_ST_ within Japan was 0.051 (0.251 maximum and −0.003 minimum), and within Taiwan 0.081 (0.157 maximum and 0.013 minimum). *F*_ST_ values within Japan decreased if the Yakushima population (0.037 mean and 0.155 maximum) and both Yakushima and Iwaki populations (0.081 mean and 0.157 maximum) were excluded. For the PCoA based on *F*_ST_, axis 1 explained 96.4% of the variance, and the Japanese and Taiwanese populations were separated (Figure 5a). Axis 2 explained 1.68% of the variance, and the Yakushima population was distinct from the other Japanese populations. The higher latitudinal populations, such as Iwaki and Yaita, fell on the lower side of axis 2, while lower latitudinal populations, such as Kobayashi and Besshiyama, fell on the upper side of axis 2 (Figure 5b). For Taiwan, the relationship between the north–south populations and its position relative to axis 2 was unclear (Figure 5c).

**TABLE 2:** Mean, median, maximum, and minimum pairwise genetic differentiation (*F*_ST_).

	Mean *F*_ST_	Median *F*_ST_	Maximum *F*_ST_	Minimum *F*_ST_
All population	0.259	0.059	0.717	−0.003
Between Japan and Taiwan	0.694	0.694	0.717	0.673
Within Japan	0.051	0.031	0.251	−0.003
Excluding Yakushima	0.037	0.028	0.155	−0.003
Excluding Yakushima and Iwaki	0.030	0.025	0.106	−0.003
Within Taiwan	0.081	0.064	0.157	0.013

**FIGURE 6:**
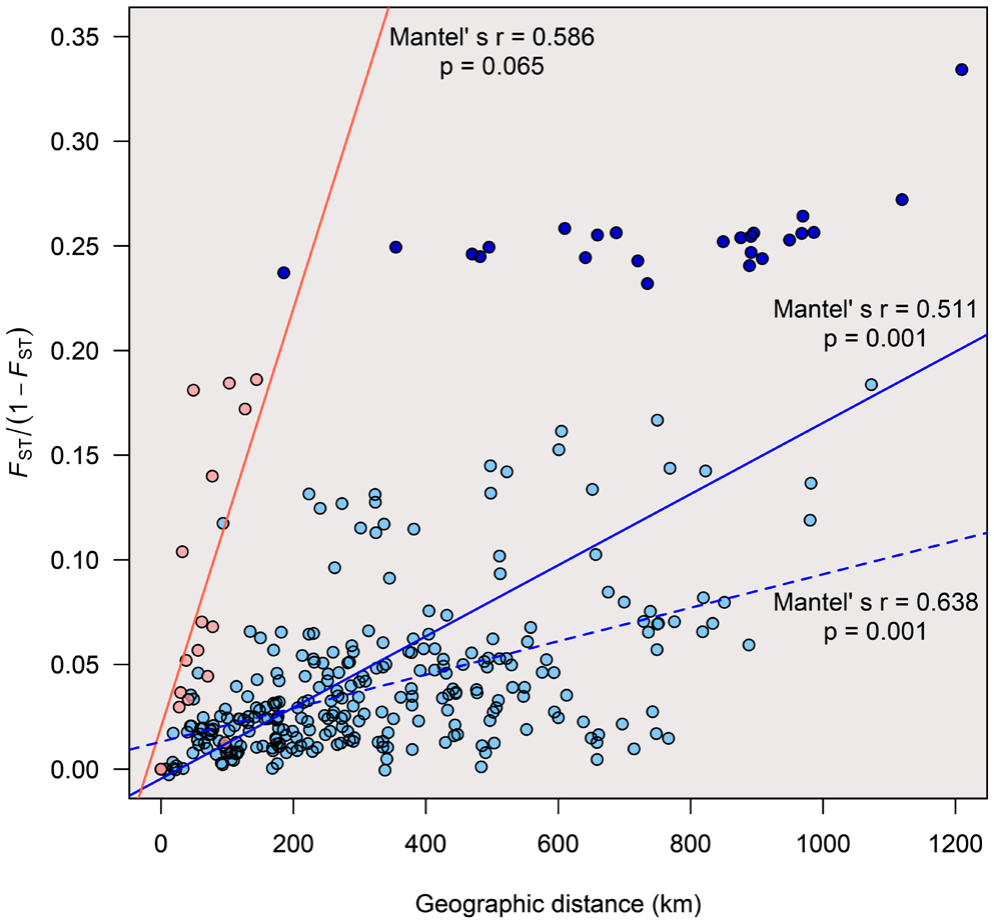
Analysis of isolation by distance (IBD) within populations of *Chamaecyparis obtusa* in Japan and Taiwan. The pairwise *F*_ST_/(1 − *F*_ST_) values are plotted against the geographical distances (km) between populations. Blue circles indicate the values among populations in Japan, while red circles indicate the values among populations in Taiwan. Darker blue circles indicate the values between the Yakushima population and other populations in Japan. The red and blue lines indicate the correlation slopes for Japan and Taiwan, respectively. The blue dashed line indicates the correlation slope for Japan excluding the Yakushima population. Mantel's r indicates the correlation coefficients between the pairwise *F*_ST_/(1 − *F*_ST_) values and the geographic distance.

The Mantel test showed a highly significant correlation between genetic divergence, denoted by pairwise *F*_ST_/(1 − *F*_ST_), and geographical distance between the Japanese populations (Mantel's *r* = 0.511, *p* = 0.001). For Taiwan, the Mantel test result was partially significant between populations (Mantel's *r* = 0.586, *p* = 0.065) (Figure 6). The correlation between pairwise *F*_ST_/(1 − *F*_ST_) and geographical distance between populations of Japan increased when the Yakushima population was excluded (Figure 6: dashed line).

### 3.3 Phylogenetic Relationships and Genetic Structure

**FIGURE 7:**
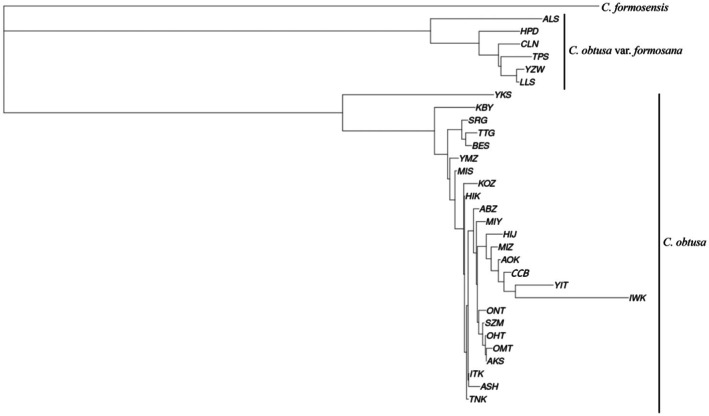
Phylogenetic relationships within populations of *Chamaecyparis obtusa*. Abbreviations for the populations are provided in Table 1. The phylogenetic relationships were inferred from BIONJ (Gascuel 1997), with algorithms to accommodate incomplete distance matrices (Criscuolo and Gascuel 2008), based on *F*_ST_ (Nei 1987).

**FIGURE 8:**
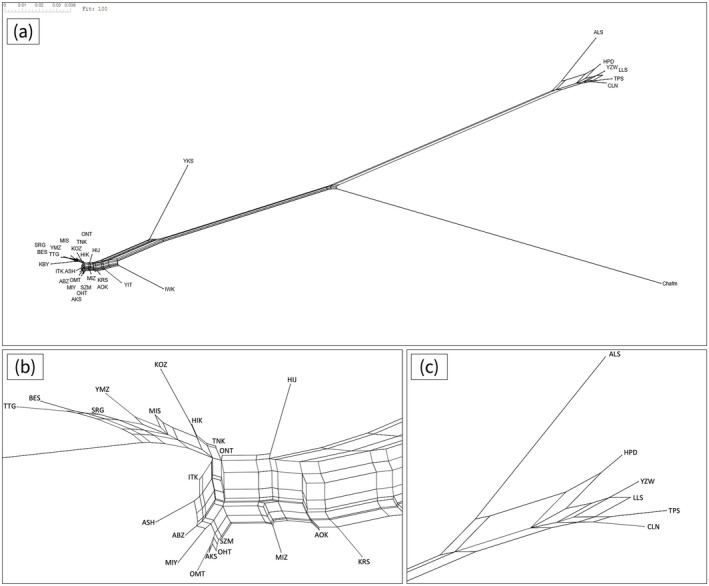
Phylogenetic relationships within populations of *Chamaecyparis obtusa* based on pairwise genetic differentiation (*F*_ST_) (Nei 1987) visualized using the SplitsTree software (Huson and Bryant 2006). (a) Whole phylogenetic tree; (b) the phylogenetic tree around mainland Japan; (c) the phylogenetic tree around Japan.

Both the NJ tree based on *F*_ST_ and maximum likelihood phylogenetic tree suggested that the populations in mainland Japan ( *C. obtusa* ) and Taiwan ( *C. obtusa* var. *formosana*) belonged to different clades (Figures 7 and S2). The southern‐most populations in Japan (Yakushima) and Taiwan (Alishan) were monophyletic in each clade in the NJ tree (Figure 7). Within Japan, the northern‐most populations (Iwaki and Yaita) had longer nodes compared with other populations. The phylogenetic relationships implemented in the software SplitTree indicated more mesh‐patterned networks existed between populations in Japan than in Taiwan (Figure 8).

**FIGURE 9:**
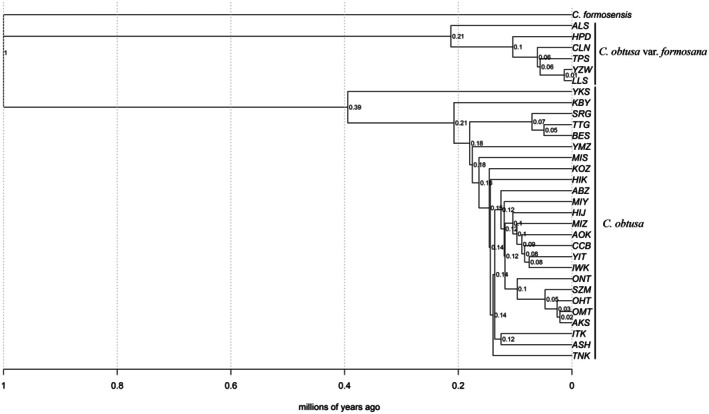
An ultrametric neighbor‐joining tree for populations of *Chamaecyparis obtusa* . Abbreviations for the populations are provided in Table 1. The divergence date based on the length of the tree using penalized and maximum likelihoods, assuming auto‐correlated substitution rates (Paradis 2013), is indicated at each node. The units are millions of years ago (Ma).

Based on the branch length of the NJ tree, the divergence time between the populations in Japan ( *C. obtusa* ) and Taiwan ( *C. obtusa* var. *formosana*) was estimated at 1 Ma (Figure 9). The Yakushima population was estimated to have diverged from other Japanese populations at 0.39 Ma. The Alishan population was estimated to have diverged from other Taiwanese populations at 0.21 Ma.

**FIGURE 10:**
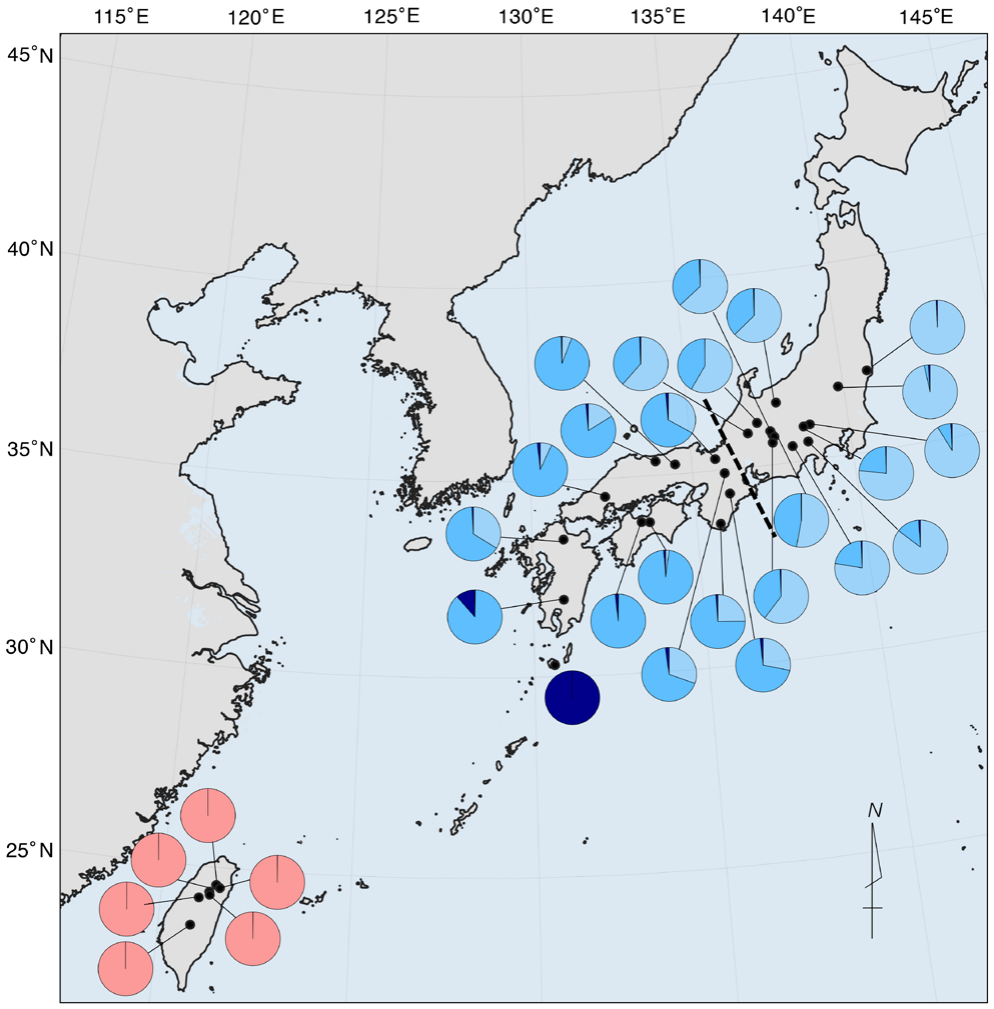
The genetic structure of *Chamaecyparis obtusa* populations. Black dots indicate the location of each population, and the pie charts indicate the posterior probability of assignment for each individual in the *K* = 4 genetic clusters identified by the software ADMIXTURE (Alexander et al. 2009). Dashed line indicates a boundary which we do not recommend seedling transfer in the mainland Japan associated with the *K* = 4 of the ADMIXTURE result.

**FIGURE 11:**
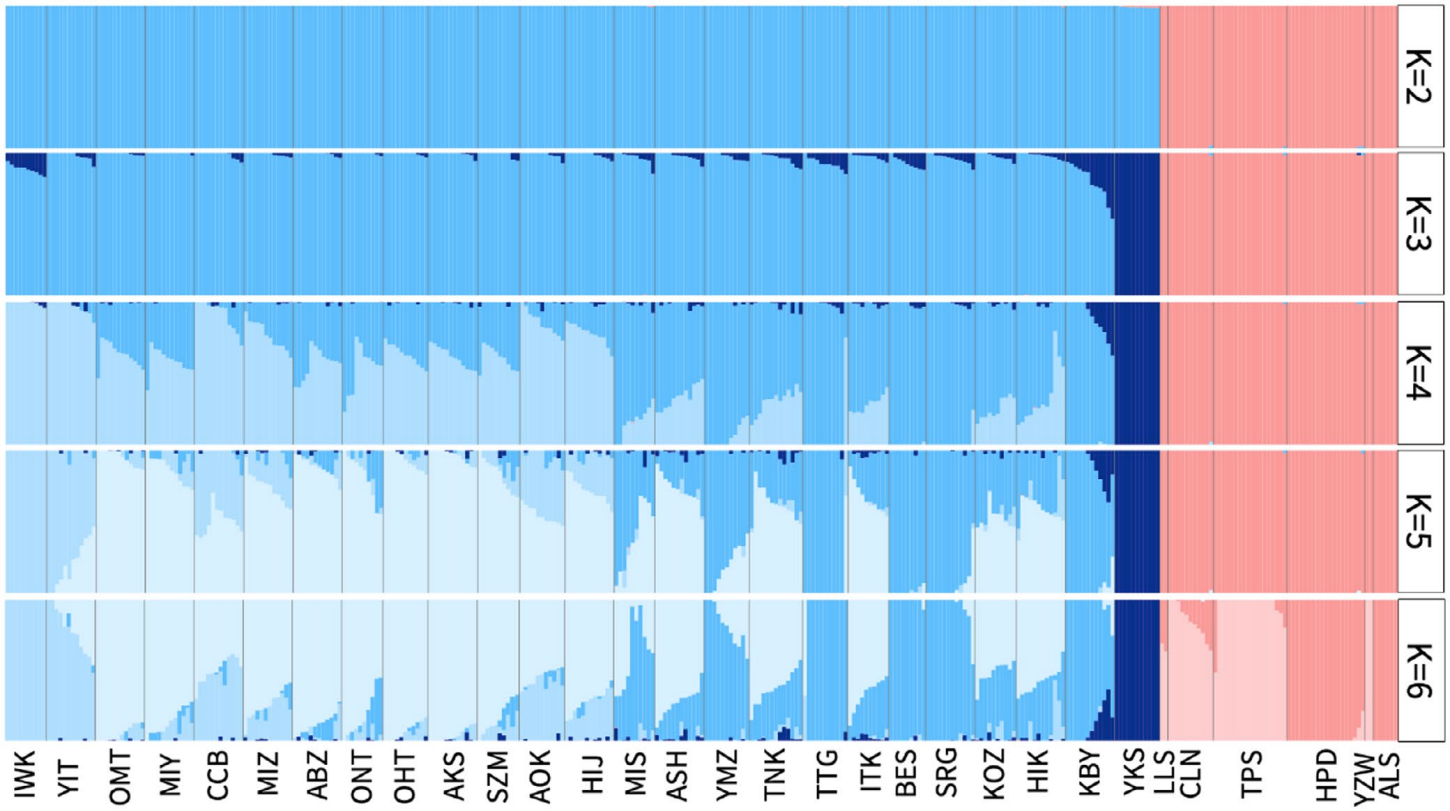
ADMIXTURE (Alexander et al. 2009) plots (*K* = 2, 3, 4, 5, 6). The names of the populations are indicated at the bottom of the plots, with higher latitudinal populations on the left side and lower latitudinal populations on the right side. Abbreviations for the populations (Table 1) are indicated at the bottom of the panel, with higher latitudinal populations on the left side and lower latitudinal populations on the right side.

The ADMIXTURE results indicated that the *K* = 4 model showed suitable clustering based on the cross‐validation procedure of each run (Figure S4). When *K* = 4, the northern populations in Japan, southern populations in Japan, and Yakushima and Taiwanese populations fell into different clusters (Figures 10 and 11). The northern populations beyond central Japan (dashed line in Figure 10) were assigned more clusters from northern Japan (the light blue cluster). When the number of *K* increased, the clusters in Japan and Taiwan did not mix (Figure 11), implying no past admixture event had occurred between them. The mixture of the Yakushima cluster into Kobayashi and other southern populations implied past secondary contacts between the southern populations in Japan (Figures 10 and 11).

### 3.4 Past Population Demography

**FIGURE 12:**
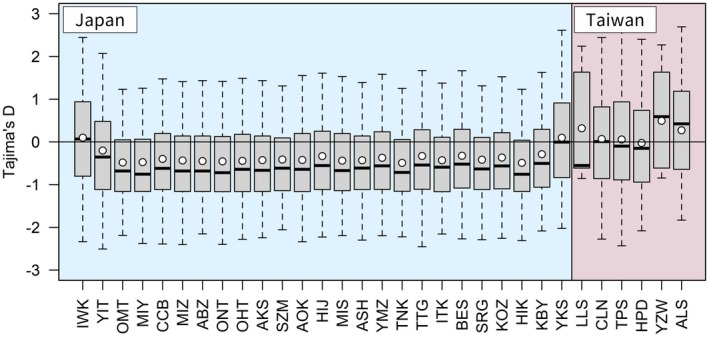
Tajima's *D* values for *Chamaecyparis obtusa* populations. White circles indicate the mean Tajima's *D* value for each population. The black horizontal bar in each box indicates the median value. Abbreviations of populations are indicated at the bottom of each panel, with higher latitudinal populations on the left side and lower latitudinal populations on the right side. Abbreviations for the populations (Table 1) are indicated at the bottom of the panel, with higher latitudinal populations on the left side and lower latitudinal populations on the right side.

The central populations in mainland Japan showed a negative mean Tajima's *D* value (Figure 12; Table 1). In Taiwan, the northern‐most and southern‐most populations (Lalashan, Yuzhuowan, and Alishan) showed positive mean Tajima's *D* values (Figure 12; Table 1).

Based on the results of the DIYABC, scenario 3, with Taiwan diverging from Japan without bottlenecks (Figure S1c), was selected as the best (130 votes with posterior probabilities of 0.449). Following scenario 3, scenario 1, with a common ancestor that diverged between Japan and Taiwan (Figure S1a), and scenario 6, with Japan diverging from Taiwan and a population size change in Japan (Figure S1f), were selected (126 and 112 votes, respectively). The estimated time of divergence between Japan and Taiwan was 26,409 generations (95% confidence interval (CI) 3922–50,705 generations), equivalent to 1.32 Ma (95% CI 0.20–2.54 Ma), based on a generation time of 50 years, during the early Pleistocene. The estimated time of divergence between Japan and Yakushima was 416 generations (95% CI 36–954 generations), equivalent to 0.02 Ma (95% CI 0.002–0.05 Ma) based on a generation time of 50 years, during the late Pleistocene.

### 3.5 Potential Distribution at Current and Last Glacial Maximum

**FIGURE 13:**
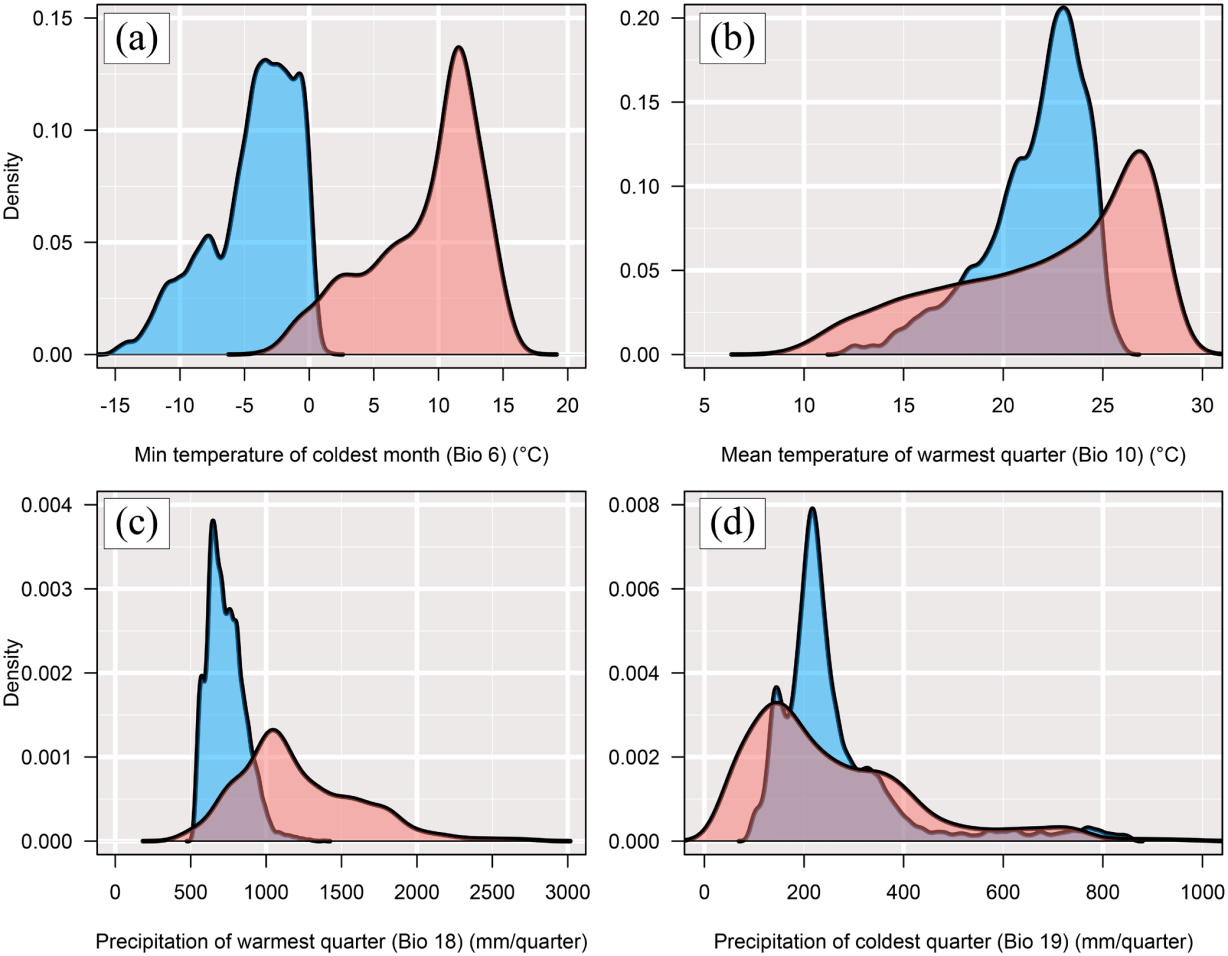
Density plots for the (a) minimum temperature of the coldest month (Bio 6) (°C), (b) mean temperature of the warmest quarter (Bio 10) (°C), (c) precipitation of the warmest quarter (Bio 18) (mm/quarter) and (d) precipitation of the coldest quarter (Bio 19) (mm/quarter) across the potential distribution range for *Chamaecyparis obtusa* in Japan (blue plots) and *Chamaecyparis obtusa* var. *formosana* in Taiwan (red plots).

The mean AUC value for the random 20% test datasets using the MaxEnt model was 0.992, categorized as “excellent” in accordance with Swets (1988). The most important variable for the model was precipitation of the warmest quarter (Bio 18: 60.2% of the overall contribution), followed by precipitation of the coldest quarter (Bio 19: 27.7%), mean daily air temperature of the warmest quarter (Bio 10: 9.5%), and minimum temperature of the coldest month (Bio 6: 2.6%) (Table 3). The potential distribution of the current dataset is represented in Figure 3b. In Taiwan, the potential distribution covered almost all of the land mass. The climatic niche conditions differed between the populations in Japan and Taiwan (Bio 18: *W* = 5,475,528, *p* < 0.001, Bio 19: *W* = 30,378,474, *p* < 0.001, Bio 10: *W* = 22,129,690, *p* < 0.001, Bio 6: *W* = 327,323, *p* < 0.001): the Taiwanese *C. obtusa* var. *formosana* habitats were characterized by warmer and wetter conditions in summer than those for the Japanese *C. obtusa* (Figure 13).

**TABLE 3:** Percentage contribution and permutation importance of the bioclimatic variables used for the distribution modeling of *Chamaecyparis obtusa* .

Variables	Abbreviations	Percentage contribution (%)	Permutation importance (%)
Precipitation of the warmest quarter	Bio 18	60.2	60.2
Precipitation of the coldest quarter	Bio 19	27.7	6.4
Mean daily mean air temperatures of the warmest quarter	Bio 10	9.5	15
Minimum temperature of the coldest month	Bio 6	2.6	18.4

The potential distribution of the species during the LGM is represented in Figure 3c (under the MIROC scenario) and 3d (under the CCSM scenario). In northern Japan, the potential LGM distributions were reduced compared with the current distribution. Fossils from around the LGM have mostly been found at locations in the lower areas of the Japanese mainland, although they have also been found in northern parts of Japan, which suggests northern refugia for *C. obtusa* (Figure 3c,d). In Taiwan, the potential LGM distributions were also smaller to some extent compared with the current distribution. The potential MH distributions (Figure S5) looked similar in range to the current potential distribution (Figure 3b).

## 4 Discussion

### 4.1 Divergence of *Chamaecyparis obtusa* in Japan and Taiwan

*Chamaecyparis* is thought to be a relict genus because fossils dating from the Paleogene and Neogene have been found across vast areas of the Northern Hemisphere (Liu et al. 2009), and it appears to have migrated across NA and EA via the BLB. *Chamaecyparis obtusa* and *C. lawsonia* diverged at 47.84 Ma during the early Eocene (Wang et al. 2022), and *C. obtusa* was distributed across the Japanese archipelago from the early Miocene (around 20 Ma), based on Cupressaceae pollen fossil records (Yamanoi 1992).

This study has revealed clear genetic differentiation between *C. obtusa* populations mainland in Japan and *C. obtusa* var. *formosana* in Taiwan, as indicated by high *F*_ST_ values (0.673–0.717; Table 2), suggesting high levels of genetic variability between them (Wright 1978). The degree of this genetic divergence seems to be consistent with previous studies regarding them as separate species (e.g., Wang et al. 2003; Liao et al. 2010). In our analysis, the divergence time was estimated to be 1 Ma based on the branch lengths of the NJ tree (Figure 9), and 1.32 Ma based on the DIYABC analysis, given a generation time of 50 years. This divergence period falls within the early Pleistocene (0.774–2.58 Ma) and corresponds roughly with the estimation of 1.3 Ma by Wang et al. (2003), the latter calculated from chloroplast DNA divergence between the two lineages. Osozawa et al. (2013) analyzed the divergence time of four insect lineages in the Ryukyu islands, which used to connect Japan and Taiwan, and demonstrated that vicariant speciation occurred at 1.55 Ma, when Ryukyu became divided into smaller island units. The divergence between *C. obtusa* in Japan and *C. obtusa* var. *formosana* in Taiwan also looks likely to have arisen as a consequence of the collapse of the land bridge connecting Japan and Taiwan during the early Pleistocene (1.2–0.8 Ma: Furukawa 1979; Kuroda 1998). The Mid‐Pleistocene Climate Transition (MPT) across 1.25–0.75 Ma led to change the cyclicity of glacial ages and increase the ice mass with a sea level decreasing (Mudelsee and Schulz 1997; Herbert 2023), as well as an increase in global aridity (Raymo et al. 1997). However, the fragmentation and landform change of the Ryukyu Islands are considered to be associated with the formation of the Ryukyu Trough and tectonic plate movements, rather than with climatic changes (Kuroda 1998), suggesting that geological processes played a significant role in the divergence of *C. obtusa* .

Our study could not determine whether *C. obtusa* in mainland Japan or *C. obtusa* var. *formosana* in Taiwan was the ancestral lineage, based on either the NJ tree (Figure 7) or the maximum likelihood phylogenetic tree (Figure S3). From the DIYABC analysis, populations in Taiwan diverging from the populations in mainland Japan were chosen as the best demographic scenario (Figure S1). Similarly, a population size decline before the Pleistocene in Taiwanese populations was inferred using the maximum likelihood method (Figure S6), suggesting that there were bottleneck events before divergence from a large ancestral population. Moreover, higher levels of genetic diversity in the Japanese populations than in the Taiwanese also agree with a Japanese origin hypothesis. Conversely, based on Tajima's *D* values, bottlenecks in the populations in Taiwan (negative mean Tajima's *D* values) were not inferred, while bottlenecks (recent population expansions) in Japanese populations were inferred (Figure 12; Table 1). Gattepaille et al. (2013) have demonstrated that the strength and timing of bottlenecks affect Tajima's *D* values, both positively and negatively. These contradictory results suggest that there was no clear large ancestral population of *C. obtusa* before the divergence between Japan and Taiwan, and that the ancestral lineage of *C. obtusa* is likely to have migrated separately into Japan and Taiwan during the early Pleistocene, when Japan and Taiwan were connected via the Chinese continent (Ota 1998). However, we believe that the ancestral population of *C. obtusa* in Japan is likely to have migrated to Taiwan via the Ryukyu islands. This has also been evidenced by previous studies (Liao et al. 2010; Huang 2014), and *C. obtusa* fossils dating from the late Miocene to early Pleistocene have been found on one of the Ryukyu islands (Okinawa Chigakukai 1982).

Within the Japanese populations, the Yakushima population appears to have diverged earlier than other populations (Figures 7, 8, 9). High levels of genetic distinctness are exhibited by the Yakushima population (Figures 5, 10 and 11), which is consistent with a previous study using simple sequence repeat (SSR) markers (Matsumoto et al. 2010). Similarly, high levels of genetic distinctness in Yakushima populations have been observed in a variety of organisms, such as *Cryptomeria japonica* (Tsumura et al. 2012, 2014, 2020) and *Cervus nippon* (Terada and Saitoh 2018). Takahashi et al. (2024) estimated the divergence times of 26 plant taxa in Yakushima from their counterparts, and 19 of them overlapped with the last glacial period (11,500–116,000 years ago). Similarly, the divergence time between the Yakushima *C. obtusa* population and other populations was estimated to be 0.02 Ma in the DIYABC analysis. Hence, the genetic divergence between Yakushima and other populations could be explained by its long geographic isolation from mainland Japan and adaptation to the local climatic conditions characterized by high rainfall (Table 1).

Similar to the Yakushima population, the Iwaki population, located at the northern‐most edge of the distribution range, exhibited high levels of genetic distinctiveness (Figures 5, 7 and 8). Both the Yakushima and Iwaki populations exhibited mean values of Tajima's *D* close to zero (Figure 12), which suggests long‐term stable persistence and, in turn, stable refugia during the glacial period. The occurrence of LGM macro‐fossils at northern locations close to Iwaki (Figure 3c,d; Nishiuchi et al. 2017) also suggests the existence of refugia around those areas. In Europe, there is much evidence of northern refugia as large glaciers and permafrost reached southern regions (e.g., Stewart and Lister 2001; Willis and van Andel 2004; Petit et al. 2003; Svenning et al. 2008). Because freezing resistance to −25°C has been reported in *C. obtusa* (Takagi 1993), it would not be unexpected if this species had refugia in the north, as in European case studies.

### 4.2 Genetic Diversity and Recent Demography

The overall low heterozygosity found in this study (overall mean *H*_O_ 0.074, overall mean *H*_E_ 0.077; Table 1) might be caused by the type of molecular methods used, that is, MIG‐seq analyses, which evaluated a large number of minor SNPs in inter‐simple sequence repeat regions. Similarly, previous studies that used MIG‐seq analysis have exhibited low heterozygosity (e.g., Onosato et al. 2021; Nishio et al. 2023; Takahashi and Suyama 2023).

Higher levels of genetic diversity in the Japanese compared with Taiwanese populations probably reflect differences in recent population demographic histories as well as the ancestral lineages, as already discussed. Narrower potential distributions in the LGM are inferred for mainland Japan (Figure 3c,d), and a recent population expansion, apart from marginal populations, is suggested by the Tajima's *D* values (Figure 12), which correspond with a population expansion and acceleration in evolutionary rates in the Cupressaceae lineage (Kusumi et al. 2002, 2010), although estimation using the maximum likelihood method did not infer population expansions in Japanese clusters (Figure S6). In addition, genetic differentiation between the populations appears to have formed in response to the geographic distances between them (Figure 6). Many networks visualized using SplitsTree software (Huson and Bryant 2006) have suggested incomplete lineage sorting among populations in Japan (Figure 8). This suggests that population expansions after the LGM and frequent gene flow between populations have caused higher levels of genetic diversity in Japan. Horizontal distances between populations were relatively closer in Taiwan compared with populations in Japan, and isolation by distance was weaker in Taiwan than in Japan (Figures 6 and 8). Previous studies have analyzed the population genetics of tree species in Japan and Taiwan, such as *Quercus glauca* and *Trochodendron aralioides*, and revealed higher genetic diversity in Taiwan than in Japan (Huang et al. 2002, 2004). We suspect that these species also have a similar genetic structure and biogeographic history to our study species, but the studies also only analyzed limited sample numbers from Japanese populations.

The marginal populations in Japan and the southernmost population in Taiwan (Iwaki in the north and Yakushima in the south of Japan, and Alishan in the south of Taiwan) showed lower levels of genetic diversity than central populations, based on heterozygosity and nucleotide diversity values (Figure 4). A similar tendency has been detected in previous CAPS and SSR studies of *C. obtusa* in Japan (Tsumura et al. 2007; Matsumoto et al. 2010). The preservation of high levels of genetic distinctness and low genetic diversity in marginal populations has also been reported in some conifer tree species in Japan (*Picea jezoensis*: Aizawa et al. 2009; *Abies firma*: Uchiyama et al. 2023; *Abies sachalinensis*: Kitamura et al. 2020; *Thuja standishii*: Worth et al. 2021). According to a hypothesis proposed by Tajima (1990), there is a correlation between migration and DNA polymorphism in local populations, that is, levels of polymorphism tend to be low when migration rates are low. The lower genetic diversity in the marginal populations is probably consistent with Tajima's theory.

### 4.3 Implications of the Results for Genetic Resources of *Chamaecyparis obtusa*

All the *C. obtusa* populations exhibited values of *F*_IS_ close to 0, which suggests random mating has occurred in natural populations of this species. Then, we suggest restricted areas of natural stands of *C. obtusa* should be conserved. Contamination of seedlings and seeds between Japan and Taiwan may reduce establishment success rates because of outbreeding depression (Lynch 1991), based on the fact that *C. obtusa* in mainland Japan and *C. obtusa* var. *formosana* in Taiwan are highly diverged not only in genetic characteristics but also in climatic niches (Figure 13). In Japan, seedling transfer of *C. obtusa* is regulated by a governmental raw (Forestry Seeds and Seedlings Act (Act No. 89 of 1970) 1970) defined mainly based on the Japanese climate, in which annual mean temperature and precipitation decrease with latitude (Tsumura 2022). We recommend that seedling transfers should follow proposed genetic guidelines based on the examined genetic structure (Tsumura and Suyama 2015; Tsumura 2022), but the conservation unit of *C. obtusa* should be revised based on the results of this study (Figure 10). We maintain that marginal populations in Japan (Iwaki in the north and Yakushima in the south) require focused conservation because of their distinct genetic characteristics, as well as the southernmost population in Taiwan (Alishan). In addition, we do not recommend seedling transfer between the northern and southern clusters in Japan (dashed line in Figure 10) associated with differences in genetic structure when the *K* = 4 (Figure 11).

## Author Contributions

**Takaki Aihara:** formal analysis (lead), investigation (equal), visualization (lead), writing – original draft (lead), writing – review and editing (equal). **Chih‐Hsin Cheng:** investigation (equal), writing – review and editing (equal). **Chiou‐Pin Chen:** investigation (equal). **Chieh‐Ting Wang:** investigation (equal). **Kentaro Uchiyama:** data curation (equal), writing – review and editing (equal). **Daiki Takahashi:** investigation (equal), writing – review and editing (equal). **Yoshihisa Suyama:** conceptualization (equal), data curation (equal), funding acquisition (equal), investigation (equal), project administration (equal). **Yoshihiko Tsumura:** conceptualization (equal), investigation (equal), project administration (equal), writing – review and editing (equal).

## Conflicts of Interest

The authors declare no conflicts of interest.

## Supporting information


**Figure S1:** Six scenarios simulated in a DIYABC random forest (Collin et al. 2021).
**FIGURE S2:** Pearson's correlation coefficient among four climatic variables for species distribution modeling.
**FIGURE S3:** Phylogenetic relationships within samples of *Chamaecyparis obtusa* inferred from an IQ‐TREE (Nguyen et al. 2015).
**FIGURE S4:** Plots of the cross‐validation errors for each ADMIXTURE run (Alexander et al. 2009).
**FIGURE S5:** The potential distribution of *Chamaecyparis obtusa* in the mid‐Holocene (MH).
**FIGURE S6:** Trajectories for the effective population sizes of *Chamaecyparis obtusa* clusters when *K* = 4 in the ADMIXTURE analysis.
**TABLE S1:** Localities of the *Chamaecyparis obtusa* samples used for the species distribution modeling.
**TABLE S2:** Macro‐fossil records for *Chamaecyparis obtusa* dated to around the last glacial maximum (LGM).

**Data S1:** ece372240‐sup‐0002‐DataS1.zip.

## Data Availability

All the raw sequencing data used for the analysis is uploaded as Supporting Information. We uploaded a different set of SNPs used for the phylogenetic analysis (‘Cobtusa.phylogenetic.vcf’: 9667 SNPs from 340 samples), genetic diversity (‘Cobtusa.vcf’: 9420 SNPs from 339 samples), and population structure (‘Cobtusa.Admixture‐input.ped’: 5458 SNPs from 339 samples).
