# Supplementary material for: The Historical Biogeography of Divergence in the Relict Cypress Chamaecyparis obtusa, and the Implications for Conservation and Management in East Asia
Source: Ecol Evol. 2025 Sep 30;15(10):e72240. doi: 10.1002/ece3.72240 (PMC12483943; doi:10.1002/ece3.72240)
Supplement: Supplementary file 1 — Figure S1: Six scenarios simulated in a DIYABC random forest (Collin et al. 2021). FIGURE S2: Pearson's correlation coefficient among four climatic variables for species distribution modeling. FIGURE S3: Phylogenetic relationships within samples of Chamaecyparis obtusa inferred from an IQ‐TREE (Nguyen et al. 2015). FIGURE S4: Plots of the cross‐validation errors for each ADMIXTURE run (Alexander et al. 2009). FIGURE S5: The potential distribution of Chamaecyparis obtusa in the mid‐Holocene (MH). FIGURE S6: Trajectories for the effective population sizes of Chamaecyparis obtusa clusters when K = 4 in the ADMIXTURE analysis. TABLE S1: Localities of the Chamaecyparis obtusa samples used for the species distribution modeling. TABLE S2: Macro‐fossil records for Chamaecyparis obtusa dated to around the last glacial maximum (LGM). [file ECE3-15-e72240-s001.docx]

(b)

(a)


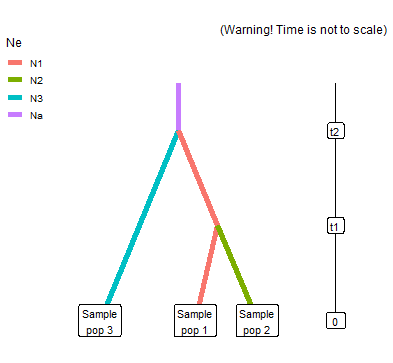

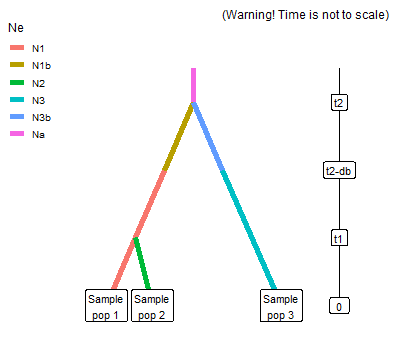


(d)

(c)


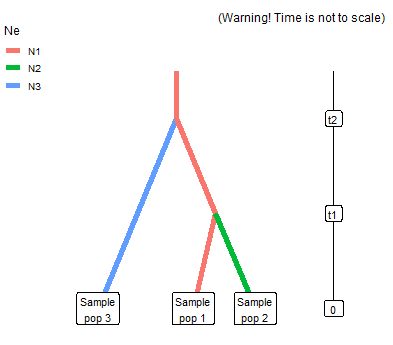

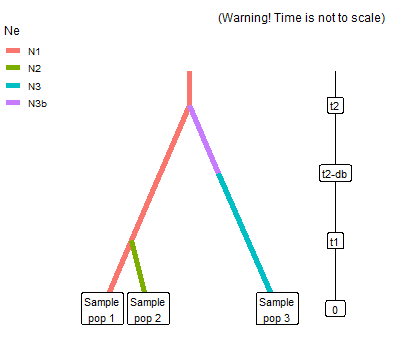


(f)

(e)


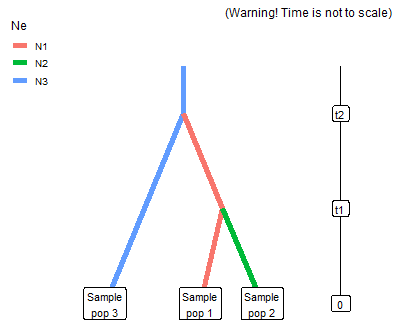

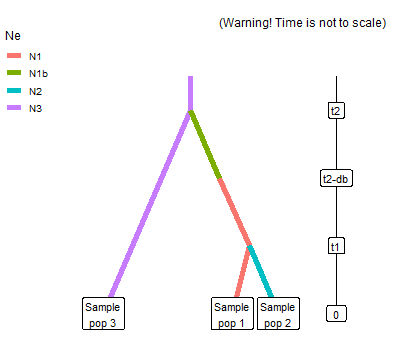


Figure S1. Six scenarios simulated in a DIYABC random forest (Collin et al., 2021).

Pop1: Japan; Pop2: Yakushima; Pop3: Taiwan. An effective population size (Ne) of each population was indicated as N1, N2 and N3. (a) Scenario 1: A common ancestor that has an effective population size, Na, that diverged between Japan and Taiwan at t2. (b) Scenario 2: Scenario 1 with population size changes in Japan and Taiwan after the divergence. (c) Scenario 3: Taiwan diverged from Japan at t2. (d) Scenario 4: Scenario 3 with population size changes in Taiwan after the divergence. (e) Scenario 5: Japan diverged from Taiwan at t2. (f) Scenario 6: Scenario 5 with population size changes in Japan after the divergence.

Figure S2. Pearson's correlation coefficient among four climatic variables for species distribution modeling.


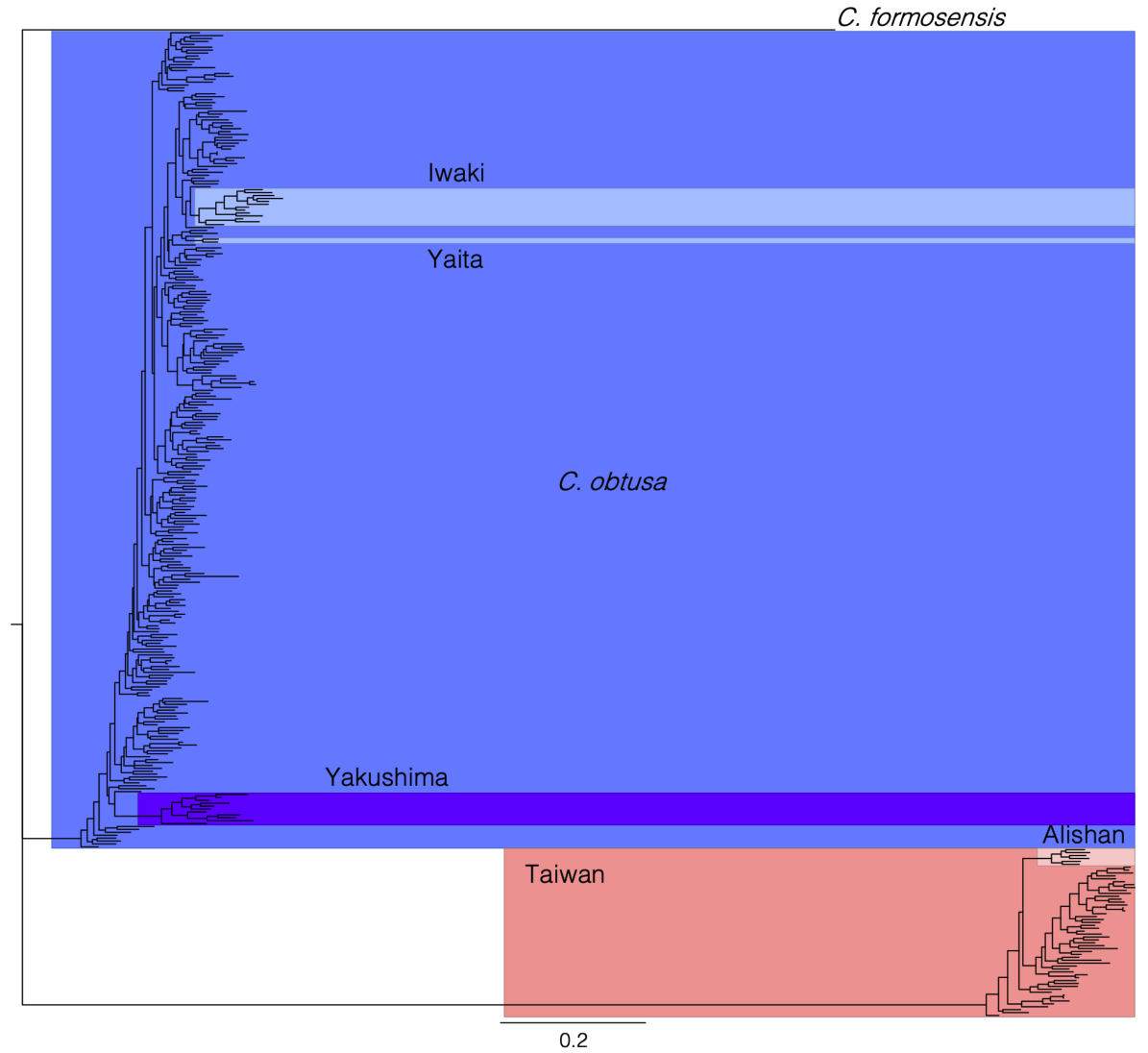


Figure S3. Phylogenetic relationships within samples of *Chamaecyparis obtusa* inferred from an IQ-TREE (Nguyen et al., 2015).


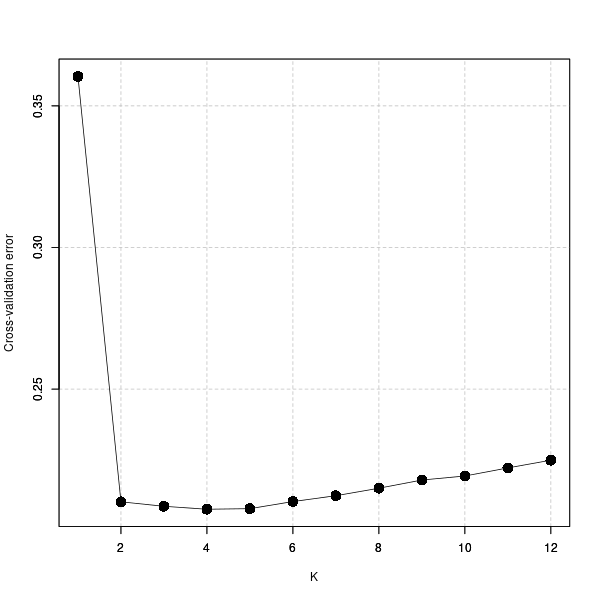


Figure S4. Plots of the cross-validation errors for each ADMIXTURE run (Alexander et al., 2009).


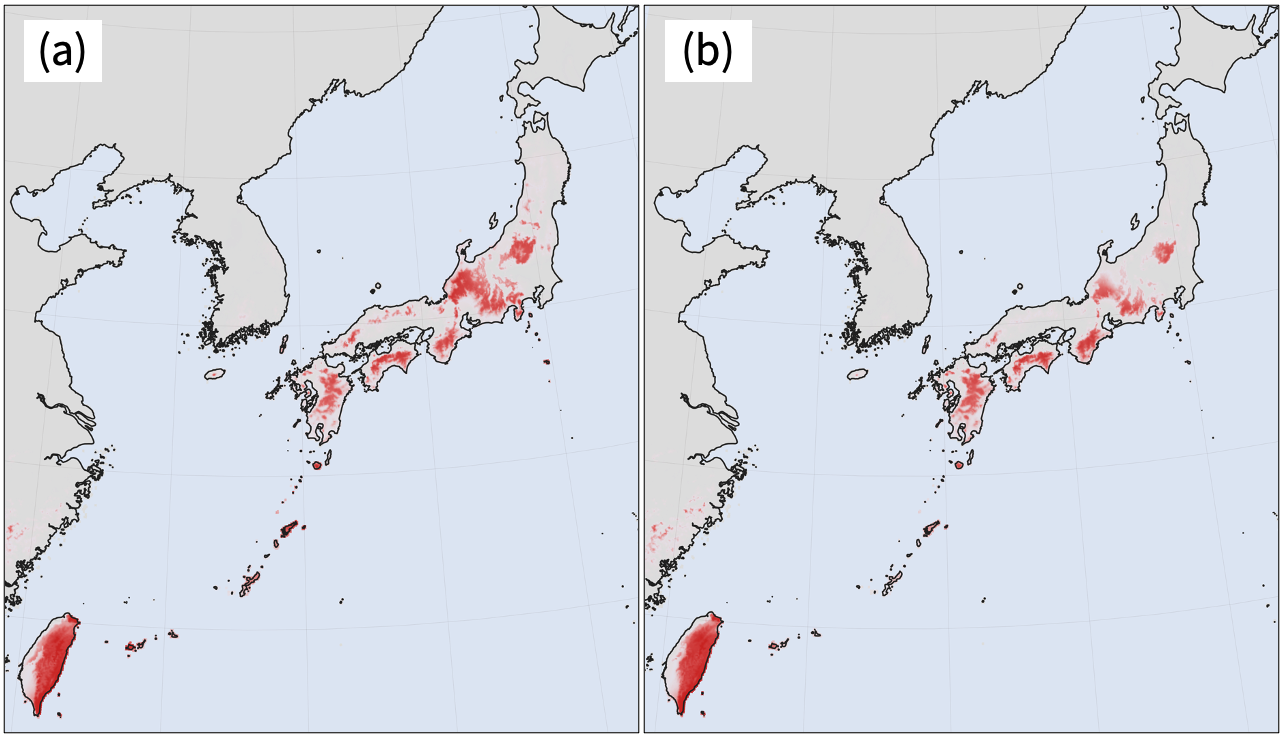


Figure S5. The potential distribution of *Chamaecyparis obtusa* in the mid-Holocene (MH).

Under (a) the MIROC-ESM scenario (Watanabe et al., 2011) and (b) the CCSM scenario (Gent et al., 2011).

Figure S6. Trajectories for the effective population sizes of *Chamaecyparis obtusa* clusters when *K*=4 in the ADMIXTURE analysis.

The light blue line indicates the results for the northern Japanese cluster (Iwaki and Yaita); the blue line indicates the results for the southern Japanese cluster (Shiragayama, Besshiyama and Tsutsuga); the dark blue line indicates the results for the Yakushima cluster; the red line indicates the results for the Taiwanese cluster.

A one-dimensional minor allele site frequency spectrum (1D-mSFS) was calculated from the vcf file with the R script “1D-msfs-R” (https://github.com/garageit46/1D-msfs-R). Using the 1D-mSFS, we estimated the trajectory of past effective population size changes with a maximum likelihood method implemented in Stairway plot v.2.1 (Liu and Fu, 2015). We used a mutation rate of 7 × 10^−9^ per site per generation, which was estimated from the divergence time between *Juniperus* and *Cupressus* (Ma et al., 2019), which are in the same family, Cupressaceae, as *Chamaecyparis*. To convert the time scale from generations to years, we assumed 50 years per generation, as in a previous study of Cupressaceae (Ma et al., 2019).

Table S1. Localities of the *Chamaecyparis obtusa* samples used for the species distribution modeling.

| No. | Latitude | Longitude |
| --- | --- | --- |
| 1 | 23.563 | 121.235 |
| 2 | 22.988 | 120.805 |
| 3 | 24.471 | 121.195 |
| 4 | 23.538 | 120.795 |
| 5 | 23.996 | 121.205 |
| 6 | 24.446 | 121.285 |
| 7 | 23.813 | 121.215 |
| 8 | 23.554 | 120.815 |
| 9 | 24.496 | 121.135 |
| 10 | 23.763 | 121.075 |
| 11 | 24.238 | 120.935 |
| 12 | 24.746 | 121.415 |
| 13 | 24.679 | 121.385 |
| 14 | 25.229 | 121.505 |
| 15 | 24.429 | 120.745 |
| 16 | 24.546 | 121.295 |
| 17 | 23.529 | 120.765 |
| 18 | 23.729 | 120.965 |
| 19 | 24.171 | 121.005 |
| 20 | 23.504 | 120.715 |
| 21 | 24.796 | 120.965 |
| 22 | 24.021 | 121.135 |
| 23 | 24.763 | 121.745 |
| 24 | 24.596 | 121.505 |
| 25 | 24.246 | 121.085 |
| 26 | 23.838 | 121.155 |
| 27 | 24.521 | 121.585 |
| 28 | 24.354 | 121.305 |
| 29 | 24.446 | 121.245 |
| 30 | 24.329 | 121.115 |
| 31 | 24.504 | 121.525 |
| 32 | 24.054 | 121.525 |
| 33 | 23.863 | 120.915 |
| 34 | 24.121 | 121.465 |
| 35 | 24.704 | 121.445 |
| 36 | 24.754 | 121.465 |
| 37 | 24.246 | 120.975 |
| 38 | 24.504 | 121.115 |
| 39 | 24.196 | 121.055 |
| 40 | 24.479 | 121.535 |
| 41 | 24.479 | 121.545 |
| 42 | 24.004 | 121.005 |
| 43 | 23.271 | 120.685 |
| 44 | 22.971 | 120.685 |
| 45 | 23.579 | 120.705 |
| 46 | 22.588 | 120.745 |
| 47 | 22.571 | 120.745 |
| 48 | 23.546 | 120.765 |
| 49 | 23.604 | 120.785 |
| 50 | 23.604 | 120.915 |
| 51 | 23.271 | 120.915 |
| 52 | 23.704 | 120.915 |
| 53 | 23.238 | 120.915 |
| 54 | 23.704 | 120.935 |
| 55 | 23.046 | 120.945 |
| 56 | 23.621 | 120.945 |
| 57 | 23.588 | 120.985 |
| 58 | 24.263 | 120.985 |
| 59 | 24.329 | 120.985 |
| 60 | 24.263 | 121.005 |
| 61 | 24.296 | 121.035 |
| 62 | 23.321 | 121.045 |
| 63 | 24.296 | 121.045 |
| 64 | 24.163 | 121.045 |
| 65 | 24.346 | 121.045 |
| 66 | 24.271 | 121.065 |
| 67 | 24.271 | 121.085 |
| 68 | 24.379 | 121.095 |
| 69 | 24.488 | 121.095 |
| 70 | 23.754 | 121.125 |
| 71 | 23.338 | 121.145 |
| 72 | 24.271 | 121.145 |
| 73 | 24.229 | 121.145 |
| 74 | 24.188 | 121.165 |
| 75 | 23.813 | 121.165 |
| 76 | 24.296 | 121.165 |
| 77 | 24.413 | 121.165 |
| 78 | 23.771 | 121.175 |
| 79 | 23.871 | 121.175 |
| 80 | 24.579 | 121.175 |
| 81 | 23.429 | 121.195 |
| 82 | 23.538 | 121.195 |
| 83 | 24.221 | 121.195 |
| 84 | 24.413 | 121.195 |
| 85 | 24.588 | 121.205 |
| 86 | 24.104 | 121.205 |
| 87 | 24.596 | 121.215 |
| 88 | 22.938 | 120.445 |
| 89 | 22.521 | 120.505 |
| 90 | 22.713 | 120.515 |
| 91 | 22.871 | 120.545 |
| 92 | 24.129 | 121.235 |
| 93 | 24.338 | 121.235 |
| 94 | 23.971 | 121.245 |
| 95 | 24.004 | 121.245 |
| 96 | 24.521 | 121.245 |
| 97 | 23.704 | 121.245 |
| 98 | 24.113 | 121.265 |
| 99 | 24.496 | 121.265 |
| 100 | 24.538 | 121.265 |
| 101 | 24.538 | 121.295 |
| 102 | 23.729 | 121.315 |
| 103 | 24.413 | 121.315 |
| 104 | 23.788 | 121.335 |
| 105 | 24.413 | 121.335 |
| 106 | 24.479 | 121.335 |
| 107 | 24.521 | 121.345 |
| 108 | 24.429 | 121.345 |
| 109 | 24.571 | 121.345 |
| 110 | 24.596 | 121.365 |
| 111 | 24.629 | 121.375 |
| 112 | 24.563 | 121.405 |
| 113 | 24.529 | 121.405 |
| 114 | 24.621 | 121.415 |
| 115 | 24.621 | 121.435 |
| 116 | 24.663 | 121.435 |
| 117 | 24.479 | 121.445 |
| 118 | 24.121 | 121.445 |
| 119 | 23.921 | 121.445 |
| 120 | 24.438 | 121.455 |
| 121 | 24.746 | 121.455 |
| 122 | 24.446 | 121.465 |
| 123 | 24.646 | 121.465 |
| 124 | 24.463 | 121.485 |
| 125 | 24.729 | 121.515 |
| 126 | 24.696 | 121.515 |
| 127 | 24.129 | 121.515 |
| 128 | 25.104 | 121.515 |
| 129 | 24.688 | 121.545 |
| 130 | 24.713 | 121.545 |
| 131 | 24.496 | 121.635 |
| 132 | 24.538 | 121.655 |
| 133 | 24.388 | 121.675 |
| 134 | 24.488 | 121.675 |
| 135 | 24.613 | 121.685 |
| 136 | 36.050 | 136.331 |
| 137 | 35.849 | 137.025 |
| 138 | 36.894 | 140.364 |
| 139 | 35.216 | 133.207 |
| 140 | 34.899 | 133.611 |
| 141 | 35.756 | 136.576 |
| 142 | 35.302 | 136.521 |
| 143 | 35.847 | 137.351 |
| 144 | 33.759 | 130.981 |
| 145 | 33.703 | 131.007 |
| 146 | 35.812 | 136.723 |
| 147 | 35.812 | 136.796 |
| 148 | 35.769 | 136.608 |
| 149 | 35.719 | 136.703 |
| 150 | 35.662 | 137.593 |
| 151 | 35.659 | 136.876 |
| 152 | 35.640 | 137.522 |
| 153 | 35.552 | 137.613 |
| 154 | 35.894 | 138.951 |
| 155 | 35.843 | 138.898 |
| 156 | 35.563 | 138.720 |
| 157 | 35.563 | 138.720 |
| 158 | 35.906 | 136.260 |
| 159 | 35.275 | 134.081 |
| 160 | 34.190 | 136.073 |
| 161 | 34.184 | 136.110 |
| 162 | 35.540 | 138.093 |
| 163 | 35.679 | 136.284 |
| 164 | 35.583 | 136.576 |
| 165 | 34.540 | 136.039 |
| 166 | 34.396 | 135.873 |
| 167 | 36.835 | 139.815 |
| 168 | 36.550 | 139.446 |
| 169 | 36.425 | 136.862 |
| 170 | 36.399 | 137.745 |
| 171 | 36.094 | 137.025 |
| 172 | 36.066 | 136.832 |
| 173 | 36.065 | 137.082 |
| 174 | 35.948 | 137.098 |
| 175 | 35.862 | 136.748 |
| 176 | 35.639 | 136.927 |
| 177 | 35.580 | 137.136 |
| 178 | 35.546 | 137.094 |
| 179 | 35.491 | 137.414 |
| 180 | 35.455 | 137.237 |
| 181 | 34.509 | 136.096 |
| 182 | 34.509 | 136.095 |
| 183 | 34.509 | 136.094 |
| 184 | 33.936 | 134.174 |
| 185 | 33.915 | 134.330 |
| 186 | 33.906 | 132.941 |
| 187 | 33.890 | 134.275 |
| 188 | 33.882 | 134.196 |
| 189 | 33.874 | 134.178 |
| 190 | 33.797 | 134.126 |
| 191 | 33.786 | 134.207 |
| 192 | 33.781 | 134.037 |
| 193 | 33.769 | 133.863 |
| 194 | 33.725 | 133.100 |
| 195 | 33.715 | 134.134 |
| 196 | 33.711 | 134.329 |
| 197 | 33.701 | 134.091 |
| 198 | 33.683 | 134.266 |
| 199 | 33.674 | 133.510 |
| 200 | 33.647 | 134.137 |
| 201 | 33.464 | 130.905 |
| 202 | 33.055 | 132.659 |
| 203 | 33.637 | 134.100 |
| 204 | 35.122 | 134.741 |
| 205 | 35.079 | 135.204 |
| 206 | 34.954 | 138.251 |
| 207 | 34.255 | 135.939 |
| 208 | 34.225 | 135.968 |
| 209 | 34.194 | 135.889 |
| 210 | 34.182 | 135.935 |
| 211 | 34.101 | 135.912 |
| 212 | 34.281 | 132.313 |
| 213 | 35.159 | 137.654 |
| 214 | 35.024 | 137.640 |
| 215 | 34.964 | 135.592 |
| 216 | 34.599 | 132.206 |
| 217 | 34.500 | 132.268 |
| 218 | 34.239 | 135.656 |
| 219 | 33.917 | 133.459 |
| 220 | 33.864 | 133.590 |
| 221 | 36.057 | 137.015 |
| 222 | 35.533 | 136.225 |
| 223 | 35.239 | 136.540 |
| 224 | 37.083 | 140.817 |
| 225 | 36.783 | 139.833 |
| 226 | 35.917 | 138.783 |
| 227 | 35.883 | 138.583 |
| 228 | 35.483 | 138.667 |
| 229 | 35.417 | 138.167 |
| 230 | 36.583 | 137.817 |
| 231 | 35.767 | 137.583 |
| 232 | 35.867 | 137.533 |
| 233 | 35.717 | 137.633 |
| 234 | 35.567 | 137.567 |
| 235 | 36.117 | 137.150 |
| 236 | 35.867 | 137.817 |
| 237 | 34.917 | 135.983 |
| 238 | 34.383 | 136.083 |
| 239 | 33.633 | 135.717 |
| 240 | 35.300 | 135.733 |
| 241 | 35.250 | 134.483 |
| 242 | 35.367 | 133.883 |
| 243 | 33.817 | 133.583 |
| 244 | 33.833 | 133.333 |
| 245 | 34.533 | 132.267 |
| 246 | 33.483 | 130.950 |
| 247 | 31.950 | 130.900 |
| 248 | 30.300 | 130.583 |

Table S2. Macro-fossil records for *Chamaecyparis obtusa* dated to around the last glacial maximum (LGM)


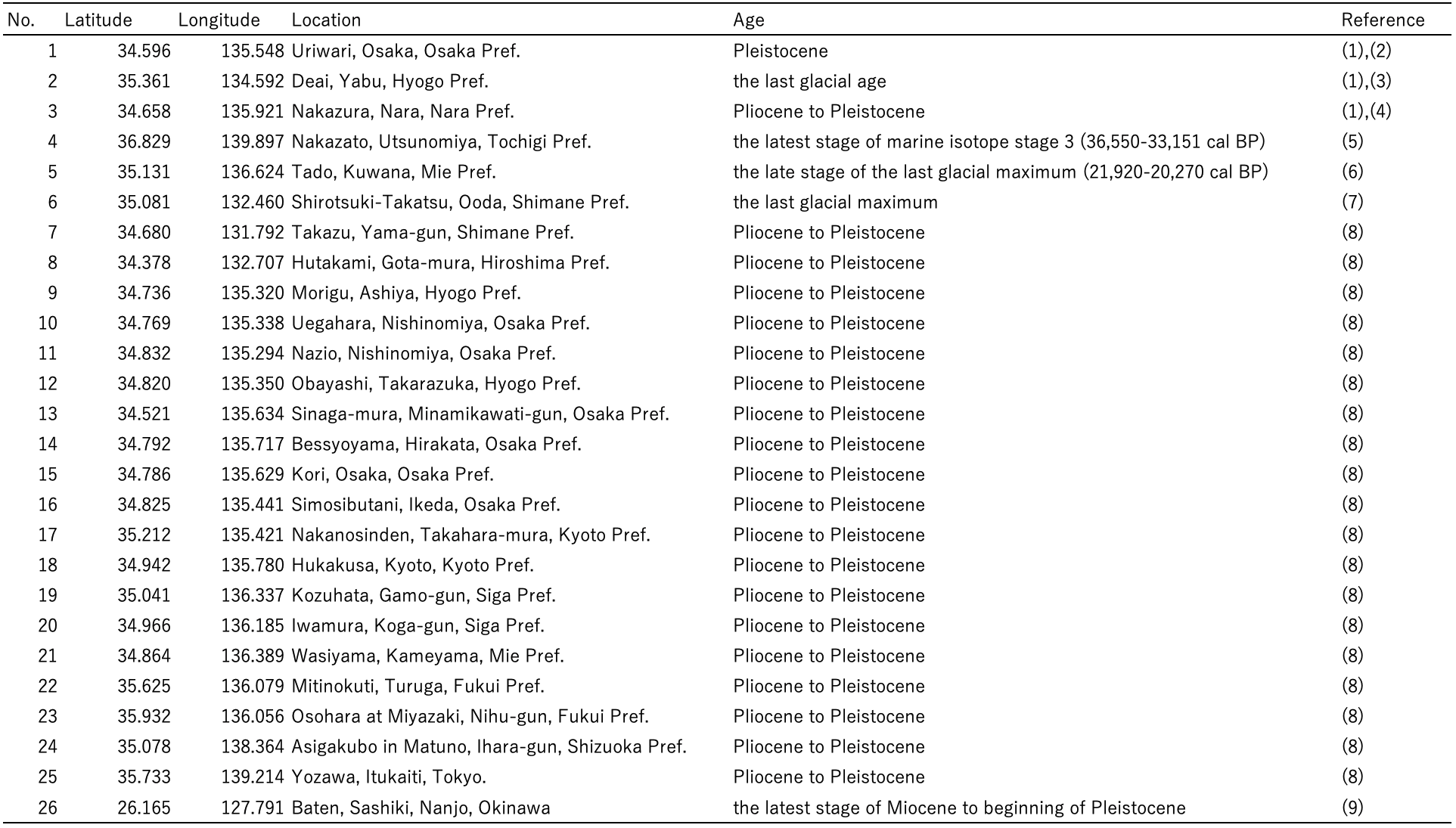


| Reference |
| --- |
| (1) Ishida I, Kudo Y, Momohara A (2016) Database of Plant Macrofossils from Archaeological Sites in Japan. Jpn J Histor Bot 24(1): 18-24 (in Japanese) |
| (2) Palynosurvey (2003) Examination of remains: Natural science analysis of the Uriwari site (UR00-11 and 01-17 surveys). In Report on the excavation survey III. Osaka city cultural properties association ed. 47-65. (in Japanese) |
| (3) Minaki M (1991) Plant macrofossils from sediments of the last glacial age in Sugigahara site. In Sugigahara site--Report on the cultural properties survey of Hyogo Pref. Education Board of Hyogo Prefecture ed. 95: 107-112. (in Japanese) |
| (4) Palynosurvey (2011) Natural science analysis of Nakazurakakinoki site. In Higasafushinda site Appendix: Nakazurakakinoki site--Report on the cultural properties survey of Nara Prefecture. 144 Archaeological institute of Kashihara ed. |
| (5) Nishiuchi, R., Momohara, A., Osato, S., Endo, K. (2017) Temperate deciduous broadleaf forest dynamics around the last glacial maximum in a hilly area in the northern Kanto district, central Japan. Quat Int 455: 113-125. |
| (6) Momohara A, Kudo Y, Miyake N, Nakamura T, Tokanai F, Tsukagoshi M (2021) Diversity of temperate flora at the Tado site, central Japan, during the last glacial stage, reconstructed from the Dr. Shigeru Miki collection. Jpn J Histor Bot 29(1): 53-68. |
| (7) Nishiuchi R, Momohara A, Tsukagoshi M (2017) Reconstruction of the last glacial maximum coniferous forest in the northeastern Chugoku District, southwestern Japan, based on macrofossil and pollen samples from the Prof. Shigeru Miki collection. Jpn J Histor Bot 26(1): 41-52 (in Japanese) |
| (8) Miki S (1958) Gymnosperms in Japan, with special reference to the remains. J Inst Polytec Osaka City Univ Ser D 9: 125-150 |
| (9) Okinawa chigakukai (1982) On the islands of Okinawa. Tsukiji shokan, Tokyo (in Japanese) |
